# Fe^III^, Cu^II^ and Zn^II^ Complexes of the Rigid 9-Oxido-phenalenone Ligand—Spectroscopy, Electrochemistry, and Cytotoxic Properties

**DOI:** 10.3390/ijms22083976

**Published:** 2021-04-12

**Authors:** Katharina Butsch, Alexander Haseloer, Simon Schmitz, Ingo Ott, Julia Schur, Axel Klein

**Affiliations:** 1 Department für Chemie, Institut für Anorganische Chemie, Universität zu Köln, Greinstraße 6, D-50939 Köln, Germany; katharinabutsch@web.de (K.B.); ahaseloe@smail.uni-koeln.de (A.H.); s.schmitz@uni-koeln.de (S.S.); 2 Institute of Medicinal and Pharmaceutical Chemistry, Technische Universität Braunschweig, Beethovenstrasse 55, D-38106 Braunschweig, Germany; ingo.ott@tu-braunschweig.de (I.O.); julia.kuschnir@gmail.com (J.S.)

**Keywords:** Iron, copper, zinc, 9-oxido-phenalenone, antiproliferative, redox

## Abstract

The three complexes [Fe(opo)_3_], [Cu(opo)_2_], and [Zn(opo)_2_] containing the non-innocent anionic ligand opo^−^ (opo^−^ = 9-oxido-phenalenone, Hopo = 9-hydroxyphenalonone) were synthesised from the corresponding acetylacetonates. [Zn(opo)_2_] was characterised using ^1^H nuclear magnetic resonance (NMR) spectroscopy, the paramagnetic [Fe(opo)_3_] and [Cu(opo)_2_] by electron paramagnetic resonance (EPR) spectroscopy. While the EPR spectra of [Cu(opo)_2_] and [Cu(acac)_2_] in dimethylformamide (DMF) solution are very similar, a rather narrow spectrum was observed for [Fe(opo)_3_] in tetrahydrofuran (THF) solution in contrast to the very broad spectrum of [Fe(acac)_3_] in THF (Hacac = acetylacetone, 2,4-pentanedione; acac^−^ = acetylacetonate). The narrow, completely isotropic signal of [Fe(opo)_3_] disagrees with a metal-centred *S* = 5/2 spin system that is observed in the solid state. We assume spin-delocalisation to the opo ligand in the sense of an opo^−^ to Fe^III^ electron transfer. All compounds show several electrochemical opo-centred reduction waves in the range of −1 to −3 V vs. the ferrocene/ferrocenium couple. However, for Cu^II^ and Fe^III^ the very first one-electron reductions are metal-centred. Electronic absorption in the UV to vis range are due to π–π* transitions in the opo core, giving Hopo and [Zn(opo)_2_] a yellow to orange colour. The structured bands ranging from 400 to 500 for all compounds are assigned to the lowest energy π−π* transitions. They show markedly higher intensities and slight shifts for the Cu^II^ (brown) and Fe^III^ (red) complexes and we assume admixing metal contributions (MLCT for Cu^II^, LMCT for Fe^III^). For both complexes long-wavelength absorptions assignable to d–d transitions were detected. Detailed spectroelectrochemical experiments confirm both the electrochemical and the optical assignments. Hopo and the complexes [Cu(opo)_2_], [Zn(opo)_2_], and [Fe(opo)_3_] show antiproliferative activities against HT-29 (colon cancer) and MCF-7 (breast cancer) cell lines in the range of a few µM, comparable to cisplatin under the same conditions.

## 1. Introduction

**Scheme 1:**
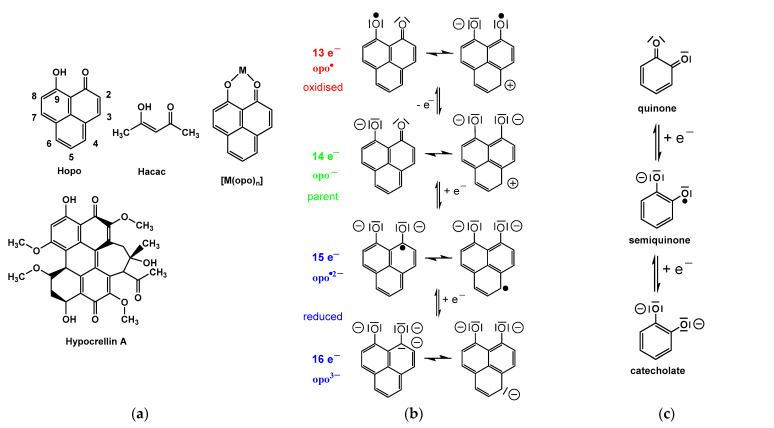
(**a**) 9-Hydroxyphenalenone (Hopo), a derivative of acetylacetone (Hacac). Both are able to form six-ring chelates when deprotonated. Structure of the cytotoxic Hypocrellin A. (**b**) Selected resonance structures of the anionic 14 valence electron system 9-oxido-phenaleneone (opo^−^) with reduced and oxidised forms. (**c**) The *o*-quinone–semiquinone–catecholate triad.

Although 9-hydroxyphenalenone (Hopo) was first synthesised 80 years ago [1], till about 10 years ago the coordination chemistry of the anionic 9-oxido-phenalenone (opo^−^) ligand (Scheme 1, left) was rather underdeveloped. Most of the reports were on the proton tautomerism [2,3,4,5,6,7,8,9] and tunnelling effects [10] of the Hopo molecule which are far more pronounced than for the corresponding parent acetyl acetone system. When coordinating deprotonated opo^−^ to metals, stable six-ring chelates stabilise the complexes and two one-electron ligand-centred reductions (opo^−^/opo^2−^ and opo^2−^/opo^3−^) and one one-electron oxidation of the ligand (opo^−^/opo) should be accessible (Scheme 1, right). The 13 e^−^ opo^•^ and the 15 e^−^ opo^•2−^ are radical species. Not surprisingly, opo complexes of the main group elements Be, B have been investigated till today towards their conducting abilities [11,12,13,14,15,16].

In contrast to the simplest β-diketonate ligand acetylacetonate (acac^−^) (Scheme 1), which has been used extensively to coordinate main group and transition metals [17,18,19,20], opo complexes are expected to be quite similar to the corresponding acac complexes, but were rather scarce till about 2010. Early reports on opo complexes of Mn^II^, Fe^III^, Co^II^, Ni^II^, Cu^II^, Zn^II^, and UO_2_^2+^ do not contain detailed characterisations or applications [21,22,23]. A number of main-group metal complexes containing B^III^ [11,12,13,14,15,16], Si^IV^ [24], Ge^IV^ [24], Be^II^ [11,21], or Al^III^ [21,25] or the f-elements Nd^III^ [25,26], Eu^III^ [25,26,27], Dy^III^ [25,28], Er^III^ [25,26], and Yb^III^ [25,26] were studied till 2010, the latter with interesting luminescence properties.

In a benchmarking study of the opo complexes of Rh^I^, Pd^II^ and Pt^II^, their cytotoxic activities against HL60 human acute myeloid leukemia cell lines were reported in 2006 to be comparable to that of cisplatin [29]. After that, the opo Pt^II^ complex [Pt(opo)(NH_3_)_2_]^+^ was studied through DFT calculations alongside other Pt^II^-containing complexes with anti-cancer activities [30], and very recently [Pt(opo)(dach)](NO_3_) (dach = 1,2-diaminocyclohexane) was found to have high antiproliferative activity in a murine NSCLC (lung cancer) tumour model [31]. This is not surprising since derivatives of Hopo [32,33] and especially the naturally occurring derivatives Hypocrellin A and B (Scheme 1) have been studied for quite some time for their cytotoxic properties [34,35,36]. Furthermore, Hypocrellin derivatives [37,38] and complexes of Zn^II^ [39], Cu^II^ [40], Co^III^ [41], Au^III^, and Pt^IV^ [42] were investigated for their photonuclease activity. Moreover, a Hypocrellin A Zn^II^ complex was reported for the optical recognition of pyrophosphate [43].

Since about 2010, researchers have recognised the enormous potential of the so-called non-innocent ligand opo^−^ with its high similarity to the *o*-semiquinones (Scheme 1) [18,44,45,46,47,48]. In complexes of non-innocent ligands, the ligands can have variable charges and oxidation states, thus making the metal oxidation state ambiguous [18]. Opo complexes of main-group and transition metals are therefore intensely studied for their magnetic, electron- and charge-transfer phenomena [44,45,46,47,48,49,50,51,52,53,54,55] as well as for their use in electroactive materials [51,52,53,54,55,56,57,58,59] or in electron transfer or related catalysis [53,55,57,60,61,62,63,64,65,66,67,68,69,70].

Herein we report on a novel facile synthesis of the simple complexes [Fe(opo)_3_], [Cu(opo)_2_], and [Zn(opo)_2_] alongside with electrochemical and spectroelectrochemical studies and their anti-proliferative properties against the cancer cell-lines HT-29 and MCF-7 We will relate our results to recent benchmarking studies in which these complexes were applied in various fields, thus giving a brief account on the interesting development the 3d transition metal complexes of the opo ligand over the last 10 years. For comparison we have also studied the acetylacetonate derivatives [Fe(acac)_3_], [Cu(acac)_2_], [Zn(acac)_2_] to probe the impact of the delocalised system of the opo ligand.

## 2. Results and Discussion

### 2.1. Determination of the pKa values of Hopo and Hacac

Hopo is completely insoluble in H_2_O and also mixtures of H_2_O and MeCN or dimethylformamide (DMF) did not allow dissolving the compound. So we embarked on determining the *pKa* values of Hopo and Hacac in MeCN solution using Kim’s voltammetric method [71]. We observed a shift peak-shift on the first reduction wave of 1,4-benzoquinone in the presence of Hopo of 192 mV in MeCN solution which translates to a *pKa* value of 20.7. The corresponding shift for Hacac was 354 mV which corresponds to a *pKa* value of 17.8. The reported *pKa* values of Hypocrellin A and Hacac in H_2_O are about 7 and 9, respectively which is in keeping with the expected slightly higher acidity of the phenol protons in Hyprocrellin A and the resonance stabilisation (see Scheme 1) compared with Hacac. In MeCN solution Hopo is less acidic than Hacac, which is probably due to the lack of stabilisation of the resonance form of opo^−^ with two negative charges on the oxygen atoms and a positive charge on the remote phenyl core (Scheme 1).

### 2.2. Synthesis and Structure Analysis of the Opo Complexes

[Fe(opo)_3_] (dark red), [Cu(opo)_2_] (brown), and [Zn(opo)_2_] (yellowish) were obtained by reacting the corresponding acac precursor complexes with Hopo (for details see Section 4) as microcrystalline solids in high yields (78–98%). The driving force of the reaction is probably in the lower solubility of the opo complexes compared with the acac derivatives, which is probably due to intermolecular π-stacking through the opo ligands in the solid state as is frequently observed for opo derivatives and complexes [14,16,56,57,62,68,72]. Elemental analyses are in line with a homoleptic composition of the three complexes as [Fe(opo)_3_], [Cu(opo)_2_] and [Zn(opo)_2_] with no other or additional ligands (= homoleptic) (see Section 4). EI–MS(+) confirmed these compositions. In solutions with coordinating solvents, [Cu(opo)_2_] and [Zn(opo)_2_] are suspected to coordinate one or two further ligands.

**Figure 1:**
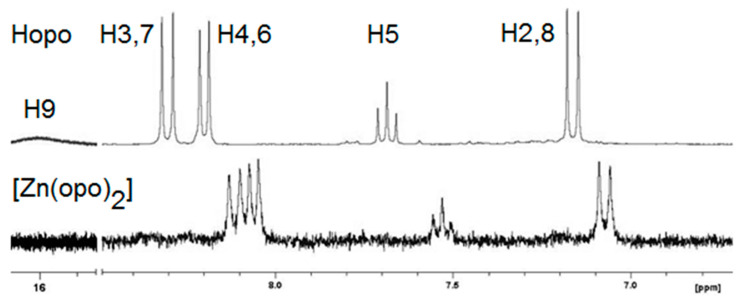
300 MHz ^1^H NMR spectra of Hopo (top) and [Zn(opo)_2_] (bottom) measured in acetone-d_6_. Assignment of Hopo protons following ref. [73].

^1^H NMR spectroscopy was possible for [Zn(opo)_2_] (Figure 1), while [Cu(opo)_2_] and [Fe(opo)_3_] are paramagnetic, so no unequivocal spectra were obtained. Compared with Hopo [73], the hydroxy proton H9 for the Zn(II) complex is missing, and a general high field-shift of all ^1^H signals upon coordination was found in agreement with data of the previously isolated complex [Zn(opo)_2_(THF)_2_] from the reaction of Hopo with ZnMe_2_ in tetrahydrofuran (THF) [49].

Our elemental analysis and MS found no evidence of solvent ligands on the isolated Zn opo complex, and the ^1^H NMR spectrum contained only traces of MeOH from the synthesis. Nevertheless, in the NMR solution, the species [Zn(opo)_2_(acetone)_2_] might be present. The shifts for [Zn(opo)_2_(THF)_2_] are 7.96, 7.89, 7.40, and 6.95 ppm, respectively, in THF–d_8_. This was very similar to our data and in line with the finding that in the crystal structure both THF ligands were found in the axial position with very long Zn‒O bonds of 2.143(1) Å [49]. Zn(opo) alkyl complexes were recently reported in the organozinc-catalysed ring-opening polymerisation (ROP) of cyclic esters [68] and intramolecular hydroamination reactions [69].

**Figure 2:**
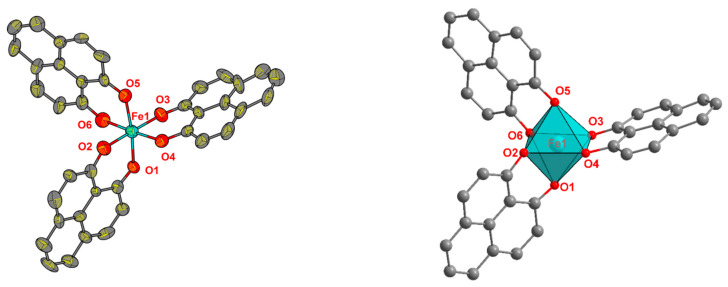
Molecular structure (left: thermal ellipsoids at 30% probability level; right: polyhedron visualisation) of [Fe(opo)_3_], H atoms omitted for clarity. Shown is the Δ configured enantiomer. The unit cell contains 3 enantiomeric pairs of the complex.

Single crystals of the dark-red complex [Fe(opo)_3_] were obtained by slow evaporation of a THF solution and submitted for an XRD study. Unfortunately, the obtained crystals were of low quality and lacked reflexes in the range of high 2θ angles. Nevertheless, the crystal structure could be solved and refined in the triclinic space group *P*-1. The crystal structure of [Fe(opo)_3_]·DMSO (DMSO = dimethyl sulfoxide) was recently published in the same space group with Z = 2 representing the two enantiomers of the complex and two co-crystallised DMSO molecules in the unit cell [57]. Our structure solution with *Z* = 6 showed three enantiomeric pairs of complex molecules, a larger volume *V* = 4779(2) to 1207.87(7) Å^3^ and the *c* axis more than double that of any axis in the structure of [Fe(opo)_3_]·DMSO. Unfortunately, our refinement parameters were quite poor with an *R*_int_ of 36% (Table S1, Supplementary Materials) which we ascribed to the poor quality of the crystal. Nevertheless, the refinement was stable and we found multiple intermolecular π-stacking in the crystal with interplanar distances ranging from 3.54 to 3.75 Å and a staggered graphite-like stacking (Figures S1–S3 in the Supplementary Materials), similar to what has been found for [Fe(opo)_3_]·DMSO [57] and other opo complexes [14,16,56,57,62,68,72]. The quality of the molecular structure of [Fe(opo)_3_], as expressed by the thermal ellipsoids and standard deviations, was good enough to assess bond distances and angles. The molecular structure of [Fe(opo)_3_] is depicted in Figure 2, and the pertinent metrical data is collected in Table S2, Supplementary Materials.

The bond distances and angles are very similar to those reported previously for [Fe(opo)_3_]·DMSO [57] and also to those of [Fe(acac)_3_] [74], proving the high similarity of the two ligands.

[Fe(opo)_3_] has recently turned out to be a very efficient catalyst for C‒H arylation [63] and was also used as cathode material for a H_2_O_2_ fuel cell together with the derivative [Fe(opo)(phen)Cl_2_] (phen = 1,10-phenanthroline) [57].

Synthesis attempts using [Ni(acac)_2_] to target the [Ni(opo)_2_] complex were unsuccessful. Yellow materials of unclear stoichiometry were obtained. Elemental analysis gave a 61.12% carbon and 3.73% hydrogen content. This lies between the values expected for [Ni(opo)_2_] (69.54% C and 3.14% H) and [Ni(acac)_2_]_n_ (45.56% C and 5.35% H) but did not fit the stoichiometry of [Ni(acac)(opo)] (61.07% C and 4.27% H). The problems with [Ni(opo)_2_] synthesis might have arisen from the structure of the precursor complex. Anhydrous [Ni(acac)_2_]_3_ is trimeric [75,76] and poorly soluble [77]. With two additional axial ligands [Ni(acac)_2_(L)_2_], the complexes (L = H_2_O, MeOH, DMF, Py, acetone) were monomeric [78,79], and the hemihydrate [Ni_2_(acac)_4_(H_2_O)_2_]·0.5H_2_O, was dimeric [80]. In addition, Ni(II) acac cluster compounds [Ni_2_Ti_2_(acac)_4_(OEt)_8_] [77] and [Ni_4_(OCH_3_)_4_(acac)_4_(CH_3_OH)_4_] [81] were reported. Such multinuclear complexes contain terminal and bridging acac ligands, the latter of which might not have been replaced, which means the reaction might have stopped half-way, leaving material with partly exchanged ligands. Leaving the reaction mixture for some time yielded polygonal green crystals which were suitable for XRD. Structure solution and refinement in the monoclinic space group *C*2/c revealed the structure of a tetranuclear complex of the formula [Ni_4_(OCH_3_)_4_(acac)_4_(CH_3_OH)_4_] (Figure S12 and Tables S3–S4 in the Supplementary Materials). This heterocubane-type compound was already reported [81] as was the phenyl derivative [Ni_4_(OCH_3_)_4_(Phacac)_4_(CH_3_OH)_4_]·½Et_2_O [82].

The complex [Ni(opo)_2_(THF)_2_] was recently synthesised from Ni(OAc)_2_·4H_2_O and Hopo and used for hydrosilylation and hydroboration catalysis [60,62].

### 2.3. EPR Spectroscopy for [Cu(opo)_2_] and [Fe(opo)_3_]

**Figure 3:**
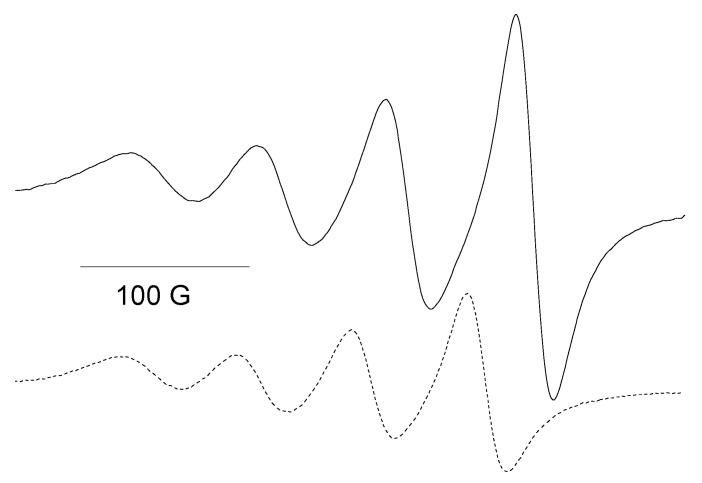
X-band EPR spectra of [Cu(opo)_2_] (solid line) and of [Cu(acac)_2_] (dashed line) both measured in DMF at 298 K at a frequency of 9.442220 GHz.

[Cu(opo)_2_] is expected to be a square planar complex, similar to [Cu(acac)_2_] [83,84,85,86,87]. In solution [Cu(acac)_2_] adds coordinating solvent molecules in the two axial positions to form hexacoordinate species [Cu(acac)_2_(L)_2_] what has been studied in detail using EPR spectroscopy [84,85,86] and we assume the same behaviour for [Cu(opo)_2_]. Very similar spectra were obtained for the opo and acac complexes (Figure 3). The complexes exhibit isotropic EPR signals with *g* values of 2.124 for [Cu(acac)_2_] and 2.127 for [Cu(opo)_2_]. The hyperfine splitting (HFS) is also slightly different with *A*_Cu_ = 76 G for [Cu(opo)_2_] and *A*_Cu_ = 67 G for [Cu(acac)_2_] (simulation for [Cu(opo)_2_] in Figure S4, Supplementary Materials). Our values recorded for [Cu(acac)_2_] are very similar to those from previous reports [84,85,86,87,88,89]. The higher HFS is in line with a lower degree of spin delocalisation from Cu^II^ over the ligand for opo^−^ compared with acac^−^.

The solid state EPR spectrum of [Fe(opo)_3_] in the X-band showed broad resonances with *g* values of about 4.5 and 2.3 (Figure S5 in the Supplementary Materials), which are in line with the reported values of 4.52 and 2.34, and the magnetic measurements are in agreement with a high-field d^5^ configuration (*µ_eff_* = 5.7 µB at 298 K) [57]. [Fe(acac)_3_] in the solid shows EPR spectra in the X-band with very similar *g* components [90,91].

**Figure 4:**
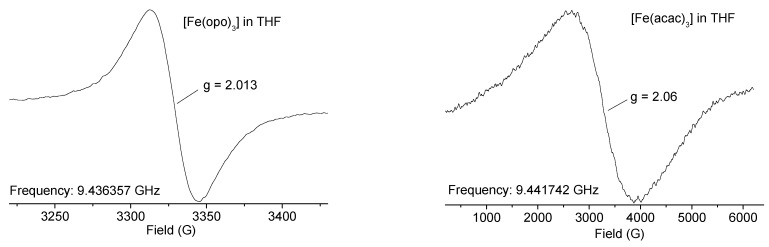
X-band EPR spectra of [Fe(opo)_3_] (**left**) and [Fe(acac)_3_] (**right**) in THF solution at 298 K.

The EPR spectrum for [Fe(opo)_3_] and [Fe(acac)_3_] in THF solution at 298 K are both isotropic but the spectral width is completely different (Figure 4). [Fe(opo)_3_] exhibits a *g* value of 2.013 and a total spectral width of about 150 G, while [Fe(acac)_3_] exhibits a *g* value of 2.060 and a spectral width of more than 5000 G. For [Fe(acac)_3_] a very similar spectrum was reported in a toluene solution at room temperature [91]. The narrow, completely isotropic signal of [Fe(opo)_3_] disagreed with a metal-centred *S* = 5/2 spin system. Either this narrow spectrum was due to marked spin delocalisation from Fe to opo^−^ or we assumed that we were observing a *S* = ½ spin system with a contribution from a diamagnetic metal, as inferred from the *g* value being only slightly higher than 2.0023 for a “free” electron in an organic molecule and from the moderate width of the signal. This would be in line with an opo^−^-to-Fe^III^ electron transfer, yielding a Fe^II^-bound opo^•^ radical complex described as [Fe^II^(opo^•^)(opo^−^)_2_].

The EPR spectrum of [B(opo)_2_]^•+^ shows HFS to protons and the B isotopes, and has a total width of about only 10 G [11]. The phenalenyl radical shows a total width of about 40 G [72] which confirms that the metal-contribution of our radical complex increased the overall spectral width, probably by broadening the lines. The reduced complex [Fe^II^(opo^−^)_3_]^−^ is assumed to have a low-spin d^6^ configuration and is thus diamagnetic [57]. Both support the idea that an opo-centred radical with *S* = ½ bound to diamagnetic Fe^II^ is observed in a THF solution.

### 2.4. Electrochemistry

**Figure 5:**
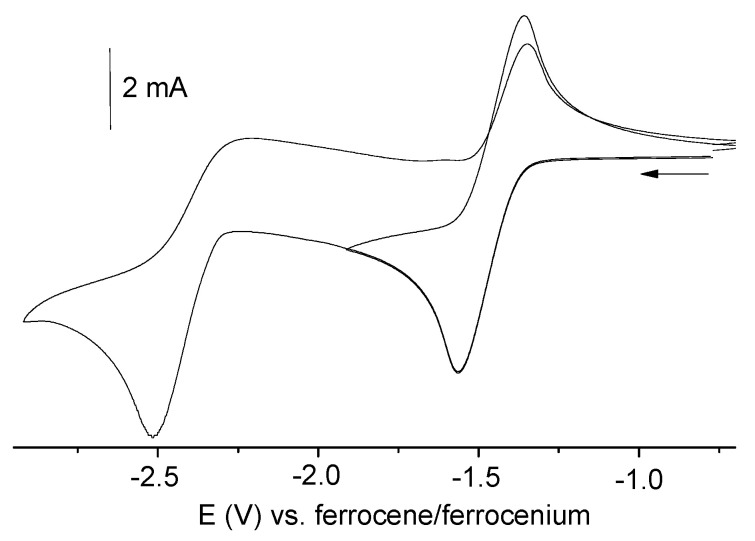
Cyclic voltammogramms of [Zn(opo)_2_] in DMF/*n*Bu_4_NPF_6_ at 298 K.

Hopo and the three Fe^III^, Cu^II^, and Zn^II^ complexes were studied through cyclic voltammetry in parallel with the corresponding acac derivatives. Hopo and the complexes can be reduced twice in the range from −1 to −2.5 V (Figure 5) in line with previous observation on Hopo and opo complexes [1,11,15,16,25,44,45,46,47,48,57,59,62,67] while oxidation was not observed in the range of 0.0 to 3.0 V (Table 1).

For the complex [Zn(opo)_2_] (Figure 4) no metal centred reduction is expected (Zn^II^ = d^10^), thus both observed reduction processes are ligand-centred and show potentials similar to those of Hopo (Figure S6, Supplementary Materials). For [Zn(acac)_2_] only one partially reversible first reduction wave was observed at a slightly more negative potential than for the opo derivative.

**Figure 6:**
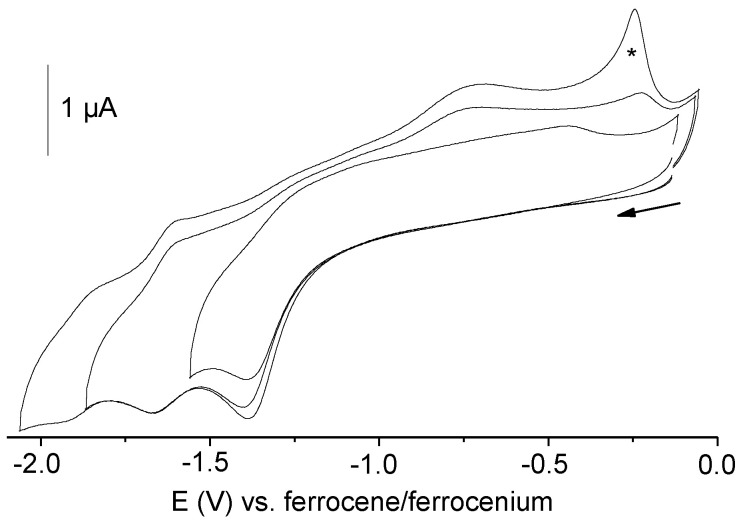
Cyclic voltammogramms of [Cu(opo)_2_] in DMF/*n*Bu_4_NPF_6_ at 298 K, * marks an adsorption process on the electrode.

For [Cu(opo)_2_] the first reductive process divides into two largely separated waves at *E*_pc_ = −1.39 V and *E_pa_* = −0.44 V which averaged to −0.92 V (Figure 6). This pair of waves represents the Cu^II^/Cu^I^ redox couple. The separation of the two processes is frequently observed for such complexes and is caused by the huge reorganisation which is necessary to change the geometry from square planar for Cu^II^ towards tetrahedral for Cu^I^ and backwards [92,93,94,95]. The same is observed for [Cu(acac)_2_] but to a much lesser extent with a separation of *E*_pc_ = −1.60 V and *E_pa_* = −0.78 V and an averaged value of −1.51 V markedly lower than for the opo derivative. Previously reported values of −1.57 V (in MeCN) and −1.61 V (in CH_2_Cl_2_) [89] fit perfectly to our measurements. As the axial positions are prone to solvent coordination, the potential of this Cu^II^/Cu^I^ couple largely varies with the solvent.

[Fe(opo)_3_] and [Fe(acac)_3_] have been studied before and the reported values agree quite well with ours [57]. The first reduction process of both complexes is assigned to a metal-centred process (Fe^III^/Fe^II^) with a slightly higher value for the acac complex indicating a slightly better σ-donation to the metal. The first opo-centred reduction of [Fe(opo)_3_] lies at −1.31 and compares to −1.41 V for the acac derivative. A second opo-centred reduction is recorded at −1.60 V. Remarkably, much more negative values of −1.83 and −2.24 V were reported for [Fe(opo)_3_] from the previous study for these processes and there seems to be a large dependence on the material of the working electrode (WE). We thus repeated our measurement using a glassy carbon (GC) WE using a Pt WE and could confirm the previous values.

### 2.5. Absorption Spectroscopy and Spectroelectrochemistry

The absorption spectra of Hopo exhibits absorptions in the range of 300 to 450 nm attributed to π−π* transitions (Table 2) [26,27,29,35,50,52]. For the Zn complex, most of the absorption maxima were almost identical to those of Hopo in keeping with the very similar electrochemical properties (Figure S7 in the Supplementary Materials). In keeping with previous reports on [Zn(acac)_2_] [96,97], we assumed that the absorbing species were the solvent-coordinated complexes [Zn(opo)_2_(solvent)_x_] with x = 1 or 2.

**Figure 7:**
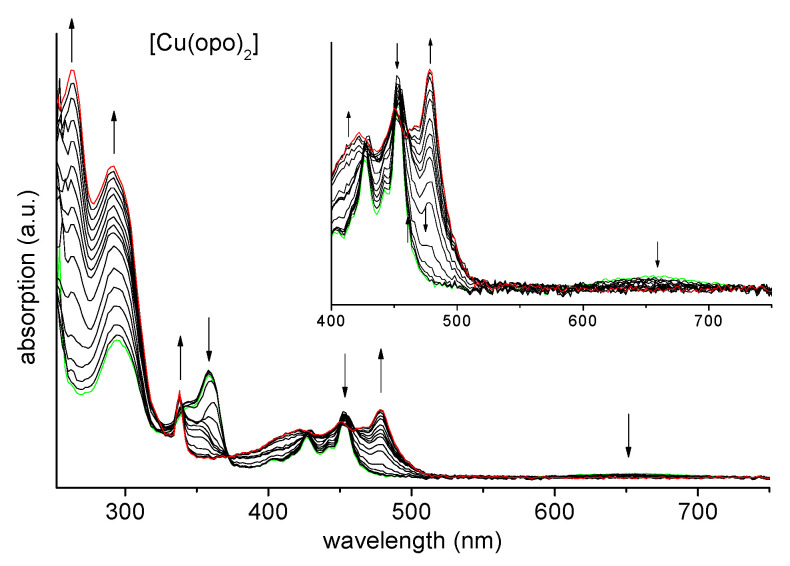
Absorption spectra of [Cu(opo)_2_] recorded during electrochemical reduction at −1.0 V in DMF/*^n^*Bu_4_NPF_6_ solution.

For the Cu^II^ complex the UV absorptions up to 380 nm are also very similar to those of Hopo (Figure 7 and Figure S8, Supplementary Materials). The structured band system peaking at 452 nm is also found for Hopo and assigned to the lowest energy π−π* transitions, but the intensity of these bands in the Cu^II^ complex is much higher (Table 2). Presumably a metal-to-ligand charge transfer (MLCT) from Cu^II^ to the lowest unoccupied molecular orbital (LUMO) of opo^−^ is admixing to these transitions in keeping with the rather high (less negative) ligand-centred reduction potentials and reports of such MLCT bands in Os^II^ and Ru^II^ complexes of opo [45,46,47,48]. For [Cu(acac)_2_] this band system is absent, supporting further the mixed π−π*/MLCT assignment. The very long-wavelength broad absorption at 651 nm is attributed to the d–d transition. For [Cu(acac)_2_] we found this absorption at 642 nm in keeping with a previous report [89]. The lower energy of the ligand field transition for [Cu(opo)_2_] translated to a weaker ligand field of the opo ligand compared with acac, in line with the poorer stabilisation of the opo^−^ anion in aprotic solvents compared with acac^−^ and the higher acidity of Hacac (vide supra). As for [Cu(acac)_2_] [84,85,86], we also assumed for [Cu(opo)_2_] a solvent-coordinated species [Cu(opo)_2_(solvent)_2_] in solution.

The spectrum of [Fe(opo)_3_] is also dominated by the π−π* transitions of the opo ligand (Figure S9, Supplementary Materials). However, the high absorption bands are markedly shifted and have gained enormously in intensity compared with the free ligand Hopo. Again, the structured π−π*(opo) band system from 400 to 500 nm is far more intense for the Fe^III^ complex and we assign this to mixed π−π*/ligand(π_opo_)-to-metal(d_Fe_) charge transfer (LMCT) transitions. A weak long wavelength absorption band is observed as a shoulder at 571 nm. Magnetic measurements [57] have previously shown that [Fe(opo)_3_] represents an Fe^III^ d^5^ high-spin system and no spin-allowed d–d transitions are thus expected. Nevertheless, this long-wavelength band might be due to a d–d transition with intensity borrowed from the close LMCT bands. The spectrum of [Fe(acac)_3_] shows an intense band at 435 in THF which can be assigned to an LMCT transition [98]. Compared with [Fe(opo)_3_] the energy of the band for the acac derivative is higher indicative for a higher lying highest occupied molecular orbital (HOMO) of the opo ligand.

Upon electrochemical reduction of [Cu(opo)_2_] the visible absorptions are red-shifted and the band at 358 nm bleaches leaving a sharp absorption at 339 nm (Figure 7). Both UV bands are markedly increased in intensity without a change in energy. We assume that the geometry re-organisation from Cu^II^ square planar (+ two solvent ligands) to tetrahedral Cu^I^ changes the probabilities for the π−π* transitions in the opo ligands, while the π−π*/MLCT bands in the visible get a red-shift from the stabilisation of the Cu HOMO (d^9^ to d^10^). The loss of the long-wavelength absorption at 651 nm additionally confirms the Cu^II^/Cu^I^ redox couple. The resulting species is best described as [Cu^I^(opo^−^)_2_]^•−^. A contribution of the resonance form [Cu^II^(opo^•2−^)(opo^−^)]^•−^ can be ruled out as a species containing opo^•2−^ is characterised by intense dark colours in the visible range of the spectrum [45,46,47,48,49,50,52,54,55,57].

**Figure 8:**
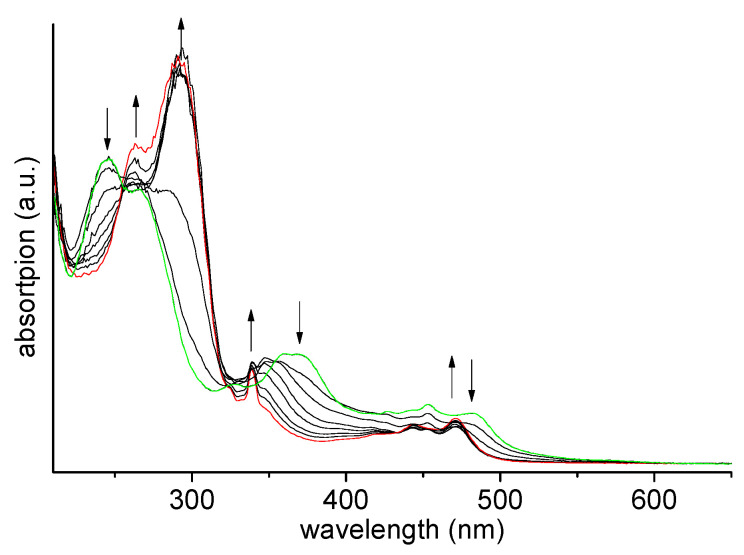
Absorption spectra of [Fe(opo)_3_] recorded during electrochemical reduction at −1.0 V in THF/*^n^*Bu_4_NPF_6_ solution.

Upon the reduction of [Fe(opo)_3_] at −1.0 V the π−π*/LMCT bands from 400 to 500 nm get a slight blue-shift and lose intensity. The band system at 360 nm bleaches leaving a sharp absorption at 339 nm (Figure 8). All absorption bands of the reduced Fe complex look very similar to those of the reduced [Cu(opo)_2_]^•−^ complex. Only the band at 264 nm is more intense than the 290 nm absorption for Cu, while for Fe these intensities are reversed. Previously, the first reduction of [Fe(opo)_3_] has been assigned to a Fe^III^/Fe^II^ couple under the assumption that in [Fe(opo)_3_]^•−^ the Fe^II^ is in a low-spin state [57]. Our experiments confirm the underlying Fe^III^/Fe^II^ couple.

As for the Cu complex, no evidence of an opo-based reduction of [Fe(opo)_3_] leading to species containing the intensely coloured radical dianion opo^•2−^ was found. Both reduced complexes clearly contained only opo^−^ ligands and Cu^I^ or Fe^II^, respectively. There was also no hint of an opo^−^-to-Fe^III^ electron transfer in the parent complex found in our UV-vis experiments, leaving the narrow EPR signal of [Fe(opo)_3_] in solution without an unequivocal explanation.

When reducing the complex [Zn(opo)_2_] at about −0.7 and at −1.3 V which both lie higher than the first reduction wave observed in CV we recorded slight shifts in the two main band systems at 350 and 450 nm. At ‒0.7 these were only slight red-shifts of both bands, while at −1.3 V the 350 nm band disappeared leaving the sharp feature at 339 nm as observed for the Cu^II^ and Fe^III^ complex after metal-centred reduction (Figure S10, Supplementary Materials). Furthermore, the long-wavelength band system shifted further to slightly lower energies. We assigned these spectroscopic features to geometry changes through the polarisation effects on the working electrode comparable to what had been observed for the reduced Cu^I^ and Fe^II^ complexes, which contained the parent opo^−^ ligand. Upon reduction at −2 V corresponding to the potential of the first reduction wave observed in the CV, the sharp 339 band and the structured absorption centred at 460 nm disappeared and broad bands at 420 and 630 nm grew in (Figure S11, Supplementary Materials). We assigned these bands to typical transitions involving a reduced opo^•2−^ ligand [45,46,47,48,49,50,52,54,55,57] and describe the complex as [Zn^II^(opo^•2−^)(opo^−^)]^•−^.

### 2.6. Antiproliferative Properties

In a benchmarking study, [Rh(opo)(CO)_2_], [Pt(opo)(NH_3_)_2_](NO_3_) and (Bu_4_N)[Pt(opo)Cl_2_] were found to have high antiproliferative activity towards human acute myeloid leukemia cells with IC_50_ values ranging from 1.7 to 3.4 µM [29]. Very recently [Pt(opo)(dach)](NO_3_) (dach = 1,2-diaminocyclohexane) was found to be active against an A549 human lung cancer cell line with activities two times higher than oxaliplatin [31]. This called for a study of Hopo and the three complexes against the common cancer cell lines HT-29 (colon cancer) and MCF-7 (breast cancer). For Hopo and the complexes, we found quite low IC_50_ values in the range of cisplatin (Table 3).

Remarkably, already the Hopo molecule shows quite high activity in line with other high antiproliferative activities of Hopo derivatives, e.g., against Leishmania [32,33]. The activities of the Fe and Zn complexes are only gradually higher but lie in the range of the reported Rh^I^, and Pt^II^ complexes [29,30,31]. While for the previously studied opo Pt^II^ complexes DNA intercalation and cisplatin-like behaviour was assumed [29,31], the apoptosis resulting from the Hopo and the Fe^III^, Cu^II^ and Zn^II^ complexes might be also caused by oxidative damage. Our spectroscopic and electrochemical findings are in line with such redox behaviour in the cell leading to reactive oxygen species, thus inducing cell damage. But in all cases, the de-coordinated ligand might be the main cause of activity and the observed differences in activity reflect the bio-availability (solubility + de-coordination rate) of Hopo or its anion. To gain more insight, we will head for the so-far unreported homoleptic Pt^II^ and Pd^II^ complexes of opo and compare their antiproliferative activities and redox chemistry (in the presence and absence of O_2_) with those of the Zn^II^, Cu^II^ and Fe^III^ derivatives reported here and study their impact on healthy and cancerous cells in further studies.

## 3. Conclusions

The three homoleptic complexes [Fe(opo)_3_], [Cu(opo)_2_], and [Zn(opo)_2_] containing the redox-active, so-called non-innocent ligand 9-oxido-phenalenone opo^−^ (Hopo = 9-hydroxyphenalenone) were synthesised from the corresponding acetylacetonates. [Zn(opo)_2_] was studied using ^1^H NMR spectroscopy, the paramagnetic [Fe(opo)_3_] and [Cu(opo)_2_] by EPR spectroscopy. While the EPR spectra of [Cu(opo)_2_] and [Cu(acac)_2_] in the DMF solution are very similar, a rather narrow spectrum was observed for [Fe(opo)_3_] in the THF solution in contrast to the very broad spectrum of [Fe(acac)_3_] in THF. The narrow, completely isotropic signal of [Fe(opo)_3_] disagreed with a metal-centred *S* = 5/2 spin system that is observed in the solid state. We assume spin-delocalisation from the Fe to the opo ligand through an opo^−^-to-Fe^III^ electron transfer. All compounds show several electrochemical reduction waves in the range of −1 to −3 V vs. the ferrocene/ferrocenium couple. While for Hopo and the Zn^II^ complex all of them are opo-centred, for Cu^II^ and Fe^III^ the very first one-electron reductions are metal-centred. Electronic absorption in the UV to vis range are due to π−π* transitions in the opo core, giving Hopo and [Zn(opo)_2_] a yellow-to-orange colour. The structured bands ranging from 400 to 500 for all compounds are assigned to the lowest energy π−π* transitions. They show markedly higher intensities and slight shifts for the Cu^II^ (brown) and Fe^III^ (red) complexes and we assume admixing metal contributions (MLCT for Cu^II^, LMCT for Fe^III^). For both complexes long-wavelength absorptions assignable to d–d transitions were detected. Detailed spectroelectrochemical experiments confirm both the electrochemical and the optical assignments but cannot find any evidence for electron transfer in the parent [Fe(opo)_3_] (described as [Fe^II^(opo^•^)(opo^−^)_2_]) as was inferred from the narrow EPR signal in solution nor for species containing the reduced opo radical opo^•2−^. UV-vis spectroelectrochemistry revealed typical absorption bands for the reduced ligand opo^•2−^ in the reduced complex [Zn^II^(opo^•2−^)(opo^−^)]^•‒^. The failure to observe these bands at 460 and 630 nm when reducing the Cu^II^ and Fe^III^ complexes confirmed the metal-centred Cu^II^/Cu^I^ and Fe^III^/Fe^II^ redox pairs. In contrast to the similar acetylacetonate ligand acac^−^, the 14-electron aromatic core of opo^−^ led to interesting optical features, while it reduced the acidity of Hopo compared with Hacac in organic solvents. Hopo and the Zn^II^, Cu^II^ and Fe^III^ complex showed high antiproliferative activity against the human cancer cell lines HT-29 and MCF-7.

## 4. Experimental Section

### 4.1. Instrumentation

NMR spectra were recorded on a Bruker Avance II 300 MHz spectrometer, using a triple resonance ^1^H, ^n^BB inverse probe head. The unambiguous assignment of the ^1^H and ^13^C resonances was obtained from ^1^H NOESY, ^1^H COSY, gradient selected ^1^H, ^13^C HSQC and HMBC experiments. All 2D NMR experiments were performed using standard pulse sequences from the Bruker pulse program library (Bruker, Rheinhausen, Germany). Chemical shifts were relative to TMS. UV-vis absorption spectra were measured on Varian Cary50 Scan (Varian Medical Systems, Darmstadt, Germany) or Shimadzu UV-3600 photo spectrometers (Shimadzu Europe, Duisburg, Germany). Elemental analyses were carried out using a HEKAtech CHNS EuroEA 3000 Analyzer (HEKAtech, Wegberg, Germany). EPR spectra were recorded in the X-band on a Bruker System ELEXSYS 500E (Bruker, Rheinhausen, Germany) equipped with a Bruker Variable Temperature Unit ER 4131VT (500 to 100 K or an Oxford Instruments helium-cryostat (300 to 4 K); the *g* values were calibrated using a dpph sample. Simulation of the EPR spectra were performed using the PEST Winsim software [100]. Electrochemical experiments were carried out in 0.1 M *^n^*Bu_4_NPF_6_ solutions using a three-electrode configuration (glassy-carbon working electrode, Pt counter electrode, Ag/AgCl pseudo reference) and an Autolab PGSTAT30 potentiostat and function generator (Metrohm, Filderstadt, Germany). The ferrocene/ferrocenium couple served as internal reference. The voltammetric determination of *pKa* values was carried out as described in the literature [71] using freshly sublimed 2,4-benzoquinone. UV-vis spectroelectrochemical measurements were performed with an optical transparent thin-layer electrochemical (OTTLE) cell [101,102].

### 4.2. Crystal Structure Determination

Measurements were performed at 293(2) K using graphite-monochromatised Mo–K_a_ radiation (*λ* = 0.71073 Å) on a IPDS I diffractometer (STOE and Cie., Darmstadt, Germany). The structures were solved by dual space methods (SHELXT-2015) [103] and refined by full-matrix least-squares techniques against (SHELXL-2017/1) [104,105]. The non-hydrogen atoms were refined with anisotropic displacement parameters without any constraints. The hydrogen atoms were included by using appropriate riding models. The numerical absorption corrections (X-RED V1.31; STOE and Cie., 2015) [106] were performed after optimising the crystal shapes using X-SHAPE V1.06 (STOE and Cie., 1999) [107]. More details on the crystal structures provided in the SI. CCDC 2071044 contain the full crystallographic data for [Fe(opo)_3_]. These data can be obtained free of charge at www.ccdc.cam.ac.uk/conts/retrieving.html (accessed on 30 March 2021) or from the Cambridge Crystallographic Data Centre, 12 Union Road, Cambridge, CB2 1EZ UK. Fax: +44-1223-336-033; Email: deposit@ccdc.cam.ac.uk.

### 4.3. Antiproliferative Activities.

The antiproliferative effects of the compounds were determined following an established procedure [99]. In short, cells were suspended in a cell culture medium (HT-29: 2850 cells/mL, MCF-7: 10000 cells/mL), and 100 mL aliquots thereof were plated in well plates and incubated at 37 °C: 5% CO_2_ for 48 h (HT-29) or 72 h (MCF-7). Stock solutions of the compounds in dimethylformamide (DMF) were freshly prepared and diluted with cell culture medium to the desired concentrations (final DMF concentration: 0.1% *v*/*v*). The medium in the plates was replaced with medium containing the compounds in graded concentrations (six replicates). After further incubation for 72 h (HT-29) or 96 h (MCF-7) the cell biomass was determined by crystal violet staining and the IC_50_ values were determined as those concentrations causing 50% inhibition of cell proliferation. Results were calculated from two independent experiments.

### 4.4. Materials and Syntheses

Water-free reactions were carried out under inert gas conditions and performed using Schlenk techniques. Solvents were dried using a MBRAUN MB SPS-800 solvent purification system. 9-Hydroxyphenalenone (Hopo) was synthesised from cinnamoyl chloride and 2-methoxynaphthalene in 92% yield following a literature procedure [108]. The acac complexes [M(acac)_2_] (M = Ni, Cu, Zn) and [Fe(acac)_3_] were synthesised as published [109].

### 4.5. Synthesis of the Opo Complexes

**[Fe(opo)_3_].** 200 mg (0.57 mmol) [Fe(acac)_3_] was ‒issolved in MeOH, a suspension of 294 mg (1.71 mmol) Hopo in MeOH was added, and the mixture was stirred at an ambient temperature for 16 h. The resulting precipitate was filtered off, washed with acetone and dried in vacuo to yield 288 mg (0.45 mmol, 78%) of a dark red solid. Elemental analyses: calc for C_39_H_21_O_6_Fe (M = 641.43 g mol^−1^) C 73.03; H 3.30; found: C 73.09; H 3.33. EI-MS(+): 641 [M]^+^, 446 [Fe(opo)_2_]^+^ *m/z*.

**[Cu(opo)_2_].** 0.50 g (1.9 mmol) [Cu(acac)_2_] was dissolved in MeOH, a suspension of 0.65 g (3.8 mmol) Hopo in MeOH was added, and the mixture was stirred at an ambient temperature for 16 h. The resulting precipitate was filtered off and washed with acetone to yield 0.82 mg (1.8 mmol, 98%) of a brown solid which was dried in vacuo. Elemental analyses: calc for C_26_H_14_O_4_Cu (M = 453.93 g mol^−1^) C 68.79; H 3.11; found: C 68.72; H 3.13. EI-MS(+): 453 [M]^+^. *m/z*.

**[Zn(opo)_2_].** 200 mg (0.76 mmol) anhydrous [Zn(acac)_2_] was suspended in MeOH. a suspension of 261 mg (1.52 mmol) Hopo in MeOH was added, and the mixture was stirred at an ambient temperature for 4 h. The resulting voluminous precipitate was filtered off, washed with acetone and dried in vacuo to yield 283 mg (0.62 mmol, 81%) of a yellowish solid. ^1^H NMR (300 MHz, acetone-d_6_): *δ* = 8.13 (d, 2H, H_2,8_), 8.07 (d, 2H, H_3,7_), 7.52 (t, 1H, H_5_), 7.08 (d, 2H, H_4,6_) ppm. Elemental analyses: calc for C_26_H_14_O_4_Zn (M = 455.78 g mol^−1^) C 68.52; H 3.10; found: C 69.07; H 3.15. EI-MS(+): 454 [M]^+^ *m/z.*

**Reaction of Hopo with [Ni(acac)_2_].** 200 mg (0.68 mmol) [Ni(acac)_2_] was dissolved in MeOH, a suspension of 234 mg (1.36 mmol) Hopo in MeOH was added, and the mixture was stirred overnight. The resulting yellow precipitate was filtered off and washed with acetone to yield 305 mg of a yellow-brown solid. Elemental analyses found: C 61.12; H 3.73.

## Figures and Tables

**Table 1:** Electrochemical properties of Hopo and opo complexes ^a.^

Compound	*E*_½_(M^n+1^/M^n^)	*E*_½_(1_ligand_)	*E*_½_(2_ligand_)
Hopo	-	−1.52	−2.51
[Fe(opo)_3_] ^b^	−1.19	−1.31	−1.60
[Fe(acac)_3_]	−1.13	−1.41	-
[Cu(opo)_2_]	−0.92 ^c^	−1.61	−1.91
[Cu(acac)_2_]	−1.19 ^d^	−1.66	−2.51
[Zn(opo)_2_]	-	−1.42	−2.50
[Zn(acac)_2_]	-	−1.51	-

^a^ Cyclic voltammetry measured in DMF/*n*Bu_4_NPF_6_ at 298 K with 100 mV/s scan rate using a glassy-carbon working electrode; potentials in V vs. ferrocene/ferrocenium. *E_1/2_* = half-wave potentials for reversible processes, *E_pc_* = cathodic peak potentials, *E_pa_* = anodic peak potentials. ^b^ Measurements using a Pt working electrode gave −1.25, −1.83, and −2.23 V. ^c^ Averaged value from *E*_pc_ = −1.39 V and *E_pa_* = −0.44 V. ^d^ Averaged value of *E*_pc_ = −1.60 V and *E_pa_* = −0.78 V.

**Table 2:** UV-vis absorption data of Hopo, opo, and acac complexes ^a.^

Compound	*ε*/nm (*ε*/Lmol^−1^cm^−1^)	Solvent
Hopo	350(1230), 393(333), 413(606), 428(504), 438(717), 451(133)	CH_3_OH
[Zn(opo)_2_]	351(1512), 393(328), 414(589), 429(626), 438(705), 453(377)	CH_3_OH
Hopo	350(1231), 393(331), 413(607), 428(502), 438(719), 451(131)	DMF
[Cu(opo)_2_]	294(8600), 358(6400), 403(1070), 426(2470), 452(3520), 651(260)	DMF
[Cu(acac)_2_]	376sh(5480), 639(1120), 642(260)	DMF
Hopo	235(950), 257(607), 265(567), 350(1230), 393(296), 414 (538), 430(397), 439(607)	THF
[Fe(opo)_3_]	265(15690), 327(4610), 361(6310), 368(6280), 453(3470), 480(2930), 571(230)	THF
[Fe(acac)_3_]	273(28449), 353(4154), 435(4193)	THF

^a^ In the case of the Cu and Zn complexes we assume the species [M(opo)_2_(solvent)_x_] to be the absorbing species as in the case for [M(acac)_2_(solvent)_x_] (x = 1 or 2) [84,85,86,96,97].

**Table 3:** Antiproliferative properties—IC_50_ values/µM.

	HT-29	MCF-7
cisplatin	7.0 ± 2 [99]	2.0 ± 0.3 [99]
Hopo	6.65 µM ± 0.70	5.11 µM ± 1.81
[Cu(opo)_2_]	10.40 µM ± 0.36	5.52 µM ± 1.06
[Fe(opo)_3_]	5.32 µM ± 0.71	4.73 µM ± 1.06
[Zn(opo)_2_]	4.17 µM ± 0.29	3.67 µM ± 1.26

## Supplementary Materials

The following are available online at https://www.mdpi.com/article/10.3390/ijms22083976/s1, Figure S1: View on the crystal structure of [Fe(opo)_3_] along the crystallographic a and b axes., Figure S2: π-stacking interactions in the crystal of [Fe(opo)_3_], Figure S3: Further π-stacking in the crystal of [Fe(opo)_3_], Figure S4: X-band EPR spectrum of [Cu(opo)_2_] in DMF at 298 K with simulation, Figure S5: X-band EPR spectrum of solid [Fe(opo)_3_] at 298 K, Figure S6: Cyclic voltammogramms of Hopo, Figure S7: UV-vis absorption spectrum of [Zn(opo)_2_] in MeOH, Figure S8: UV-vis absorption spectrum of [Cu(opo)_2_] in DMF, Figure S9: UV-vis absorption spectrum of [Fe(opo)_3_] in DMF, Figure S10: Absorption spectra of [Zn(opo)_2_] recorded during reduction, Figure S11: Absorption spectra of [Zn(opo)_2_] recorded during reduction, Table S1: Crystallographic and structure refinement data of [Fe(opo)_3_], Table S2: Selected metrical data of [Fe(opo)_3_], Figure S12: ORTEP-representations of [Ni_4_(OCH_3_)_4_(acac)_4_(CH_3_OH)_4_], Table S3: Selected distances and angles of the two cluster type compounds, Table S4: FIR vibration frequencies for the nickel cluster and [Ni(acac)_2_].

## References

[1] Koelsch C.F., Antes J.A. (1941). Studies in the perinaphthalene series. IV. Some attempts to synthesize 9-phenyl-perinaphthanone-7. J. Org. Chem..

[2] Jackman L.M., Trewella J.C., Haddon R.C. (1980). Studies in Nuclear Magnetic Resonance Spectroscopy. 17. Deuteron Quadrupole Coupling Constants in Intramolecularly Hydrogen Bonded Systems. J. Am. Chem. Soc..

[3] Rossetti R., Rayford R., Haddon R.C., Brus L.E. (1981). Proton Localization in an Asymmetric Double-Minimum Potential: 2-Methyl-9-hydroxyphenalenone. J. Am. Chem. Soc..

[4] Kunze K.L., de la Vega J.R. (1984). Intramolecular Proton Exchange in 9-Hydroxyphenalen-1-one and Methyl-9-hydroxyphenalen-1-one. J. Am. Chem. Soc..

[5] Engdahl C., Gogoll A., Edlund U. (1991). Long-Range Deuterium Isotope Effects on ^13^C NMR Shifts of Intramolecularly Hydrogen-Bonded 9-Hydroxyphenalen-1-ones. Magn. Reson. Chem..

[6] Nakai H., Sodeyama K. (2003). Energy density analysis (EDA) of proton transfer reactions in malonaldehyde, tropolone, and 9-hydroxyphenalenone. J. Mol. Struct. (Theochem.).

[7] Wang D.-P., Chen S.-G., Chen D.-Z. (2004). Theoretical studies of conjugate and substituent effects on the intramolecular proton transfer: An HF/CIS study. J. Photochem. Photobiol. A.

[8] Kuwahara D., Koyano H., Manaka T., Nakamura H., Mochida T., Sugawara T. (2006). Dynamics of 9-Hydroxyphenalenone Studied by One-Dimensional Solid-State Spin Exchange NMR. J. Phys. Chem. A.

[9] Müller C., Schroeder J., Troe J. (2006). Intramolecular Hydrogen Bonding in 1,8-Dihydroxyanthraquinone, 1-Aminoanthraquinone, and 9-Hydroxyphenalenone Studied by Picosecond Time-Resolved Fluorescence, Spectroscopy in a Supersonic Jet. J. Phys. Chem. B.

[10] Weihrich R., Limage M.H., Parker F., Fillaux F. (2004). Proton tunnelling in the intramolecular hydrogen bond of 9-Hydroxyphenalenone. J. Mol. Struct..

[11] Haddon R.C., Chichester S.V., Marshall J.H. (1986). Electron Delocalization in 9-Oxidophenalenone Complexes of Boron and Beryllium. Tetrahedron.

[12] Chi X., Itkis M.E., Kirschbaum K., Pinkerton A.A., Oakley R.T., Cordes A.W., Haddon R.C. (2001). Dimeric Phenalenyl-Based Neutral Radical Molecular Conductors. J. Am. Chem. Soc..

[13] Chi X., Itkis M.E., Tham F.S., Oakley R.T., Corders A.W., Haddon R.C. (2003). Synthesis, Structure, and Physical Properties of a New Phenalenyl-Based Neutral Radical Crystal: Correlation between Structure and Transport Properties in Carbon-Based Molecular Conductors. Int. J. Quantum Chem..

[14] Mandal S.K., Samanta S., Itkis M.E., Jensen D.W., Reed R.W., Oakley R.T., Tham F.S., Donnadieu B., Haddon R.C. (2006). Resonating Valence Bond Ground State in Oxygen-Functionalized Phenalenyl-Based Neutral Radical Molecular Conductors. J. Am. Chem. Soc..

[15] Pal S.K., Itkis M.E., Tham F.S., Reed R.W., Oakley R.T., Donnadieu B., Haddon R.C. (2007). Phenalenyl-Based Neutral Radical Molecular Conductors: Substituent Effects on Solid-State Structures and Properties. J. Am. Chem. Soc..

[16] Chi X., Itkis M.E., Patrick B.O., Barclay T.M., Reed R.W., Oakley R.T., Cordes A.W., Haddon R.C. (1999). The First Phenalenyl-Based Neutral Radical Molecular Conductor. J. Am. Chem. Soc..

[17] Stary J., Liljenzin J.O. (1982). Critical evaluation of equilibrium constants involving acetylacetone and its metal chelates. Pure Appl. Chem..

[18] Kaim W. (2019). Chelate rings of different sizes with non-innocent ligands. Dalton Trans..

[19] Vigato P.A., Peruzzo V., Tamburini S. (2009). The evolution of β-diketone or β-diketophenol ligands and related complexes. Coord. Chem. Rev..

[20] Hynes M.J. (1990). Reactions of of β-diketone complexes in solution. Rev. Inorg. Chem..

[21] Demura Y., Kawato T., Kanatomi H., Murase I. (1975). Metal Chelates of 9-Hydroxy-1-phenalenone. Bull. Chem. Soc. Jpn..

[22] Neidlein R., Behzadi Z. (1976). Synthesis and properties of some metal chelates of 9-hydroxyphenalen-1-one. Chemiker-Zeitung.

[23] Hofmann M., Musso H. (1990). Synthesis of metal complexes with bis(phenalenyl)imine ligands. Liebigs Ann..

[24] Pal S.K., Tham F.S., Reed R.W., Oakley R.T., Haddon R.C. (2005). Synthesis and characterization of germanium (IV) and silicon (IV) complexes derived from 9-hydroxyphenalenone: X-ray crystal and molecular structure of tris-(9-oxophenalenone)-germanium (IV) and silicon (IV) salts. Polyhedron.

[25] Zeng Z., Zhou J., Zhang Y., Qiao R., Xia S., Chen J., Wang X., Zhang B. (2007). Photodynamic Properties of Hypocrellin A, Complexes with Rare Earth Trivalent Ions:  Role of the Excited State Energies of the Metal Ions. J. Phys. Chem. B.

[26] Van Deun R., Fias P., Nockemann P., Van Hecke K., Van Meervelt L., Binnemans K. (2006). Visible-Light-Sensitized Near-Infrared Luminescence from Rare-Earth Complexes of the 9-Hydroxyphenalen-1-one Ligand. Inorg. Chem..

[27] Van Deun R., Nockemann P., Fias P., Van Hecke K., Van Meervelt L., Binnemans K. (2005). Visible light sensitisation of europium(III) luminescence in a 9-hydroxyphenal-1-one complex. Chem. Commun..

[28] Lan Y., Magri A., Fuhr O., Ruben M. (2016). Phenalenyl-based mononuclear dysprosium complexes. Beilstein J. Nanotechnol..

[29] Mochida T., Torigoe R., Koinuma T., Asano C., Satou T., Koike K., Nikaido T. (2006). Platinum-Group Chelate Complexes with 9-Hydroxyphenalenone Derivatives: Synthesis, Structures, Spectroscopic Properties and Cytotoxic Activities. Eur. J. Inorg. Chem..

[30] Kokoschka M., Galgonek J., Vondrasek J., Hobza P. (2016). Computational methods for the description of pharmacologically relevant platinum complexes—Molecular structure and bond dissociation. Phys. Chem. Chem. Phys..

[31] Dutta P.K., Sharma R., Kumari S., Dubey R.D., Sarkar S., Paulraj J., Vijaykumar G., Pandey M., Sravanti L., Samarla M. (2019). A safe and efficacious Pt(II) anticancer prodrug: Design, synthesis, in vitro efficacy, the role of carrier ligands and in vivo tumour growth inhibition. Chem. Commun..

[32] López-Arencibia A., Bethencourt-Estrell C.J., Freijo M.B., Reyes-Batlle M., Sifaoui I., Nicolás-Hernández D.S., McNaughton-Smith G., Lorenzo-Morales J., Abad-Grillo T., Pinero J.E. (2020). New phenalenone analogues with improved activity against *Leishmania* species. Biomed. Pharmacother..

[33] Freijo M.B., Lopez-Arencibia A., Pinero J.E., McNaughton-Smith G., Abad-Grillo T. (2018). Design, synthesis and evaluation of amino-substituted 1*H*-phenalen-1-ones as anti-leishmanial agents. Eur. J. Med. Chem..

[34] O’Brien E.M., Morgan B.J., Mulrooney C.A., Carroll P.J., Kozlowski M.C. (2010). Perylenequinone Natural Products: Total Synthesis of Hypocrellin A. J. Org. Chem..

[35] Ma G., Khan S.I., Jacob M.R., Tekwani B.L., Li Z., Pasco D.S., Walker L.A., Khan I.A. (2004). Antimicrobial and Antileishmanial Activities of Hypocrellins A and B. Antimicrob. Agents Chemother..

[36] Diwu Z., Zimmermann J., Meyers T., Lown J.W. (1994). Design, Synthesis and Investigation of Mechanisms of Action of Novel Protein Kinase C Inhibitors: Perylenequinoid Pigments. Biochem. Pharmacol..

[37] Niu T., Tian Y., Shi Y., Guo G., Tong Y., Wang G. (2021). Antifibrotic effects of Hypocrellin A combined with LED red light irradiation on keloid fibroblasts by counteracting the TGF-β/Smad/autophagy/apoptosis signalling pathway. Photodiag. Photodyn. Ther..

[38] Ding Y., Liu W., Wu J., Zheng X., Ge J., Ren H., Zhang W., Lee C.-S., Wang P. (2020). Near-Infrared Hypocrellin Derivatives for Synergistic Photodynamic and Photothermal Therapy. Chem.-Asian J..

[39] Tian C., Xu S., Chen S., Shen J., Zhang M., Shen T. (2001). Chelation of Hypocrellin B with Zinc Ions with Electron Paramagnetic Resonance (EPR) Evidence of the Photodynamic Activity of the Resulting Chelate. Free Radic. Res..

[40] Sun Y., Hou Y.-J., Zhou Q.-X., Lei W.-H., Chen J.-R., Wang X.-S., Zhang B.-W. (2010). Dinuclear Cu(II) Hypocrellin B Complexes with Enhanced Photonuclease Activity. Inorg. Chem..

[41] Sun Y., Hou Y.-J., Zhou Q.-X., Chen J.-R., Zhang B.-W., Wang X.-S. (2011). A new Co(III)-hypocrellin B complex with enhanced photonuclease activity. J. Inorg. Biochem..

[42] Zhou L., Ge X., Liu J., Zhou J., Wei S., Li F., Shen J. (2013). Internal heavy atom effect of Au(III) and Pt(IV) on hypocrellin A for enhanced in vitro photodynamic therapy of cancer. Bioorg. Med. Chem. Lett..

[43] Zhao X., Huang C.Z. (2010). A molecular logic gate for the highly selective recognition of pyrophosphate with a hypocrellin A-Zn(II) complex. Analyst.

[44] Kaim W., Lahiri G.K. (2019). The coordination potential of indigo, anthraquinone and related redox-active dyes. Coord. Chem. Rev..

[45] Hazari A.S., Paretzki A., Fiedler J., Zalis S., Kaim W., Lahiri G.K. (2016). Different manifestations of enhanced π-acceptor ligation at every redox level of [Os(9-OP)L_2_]^n^, n = 2+, +, 0, − (9-OP^−^ = 9-oxidophenalenone and L = bpy or pap). Dalton Trans..

[46] Agarwala H., Scherer T.M., Mobin S.M., Kaim W., Lahiri G.K. (2014). Bidirectional non-innocence of the β-diketonato ligand 9-oxidophenalenone (L^−^) in [Ru([9]aneS_3_)(L)(dmso)]^n^, [9]aneS_3_ = 1,4,7-trithiacyclononane. Dalton Trans..

[47] Das A., Scherer T.M., Mondal P., Mobin S.M., Kaim W., Lahiri G.K. (2012). Experimental and DFT Evidence for the Fractional Non-Innocence of a β-Diketonate Ligand. Chem.-Eur. J..

[48] Das A., Scherer T.M., Mobin S.M., Kaim W., Lahiri G.K. (2012). 9-Oxidophenalenone: A Noninnocent β-Diketonate Ligand?. Inorg. Chem..

[49] Mukherjee A., Samuel P.P., Schulzke C., Mandal S.K. (2014). Main group chemistry of 9-hydroxophenalenone: Syntheses and structural characterization of the alkaline earth and zinc complexes. J. Chem. Sci..

[50] Lehner P., Staudinger C., Borisov S.M., Klimant I. (2014). Ultra-sensitive optical oxygen sensors for characterization of nearly anoxic systems. Nat. Commun..

[51] Yakuschenko I.K., Kaplunov M.G., Krasnikova S.S. (2014). Some Metal Complexes of 9-Hydroxyphenalenone as Novel Electron Transporting Materials for OLEDs. Mol. Cryst. Liq. Cryst..

[52] Bag P., Itkis M.E., Stekovic D., Pal S.K., Tham F.S., Haddon R.C. (2015). Band Structure Engineering by Substitutional Doping in Solid-State Solutions of [5-Me-PLY(*O,O*)]_2_B_(1−x)_Be_x_ Radical Crystals. J. Am. Chem. Soc..

[53] Mukherjee A., Sau S.C., Mandal S.K. (2017). Exploring Closed-Shell Cationic Phenalenyl: From Catalysis to Spin Electronics. Acc. Chem. Res..

[54] Stekovic D., Bag P., Shankhari P., Fokwa B.P.T., Itkis M.E. (2019). Effect of Substitution on the Hysteretic Phase Transition in a Bistable Phenalenyl-Based Neutral Radical Molecular Conductor. Chem. Eur. J..

[55] Ahmed J., Datta P., Das A., Jomy S., Mandal S.K. (2021). Switching between mono and doubly reduced odd alternant hydrocarbon: Designing a redox catalyst. Chem. Sci..

[56] Dey S.K., Honecker A., Mitra P., Mandal S.K., Mukherje A. (2012). Magnetostructural Studies on Tetranuclear Manganese [Mn^III^_2_Mn^II^_2_] Complexes of 9-Hydroxyphenalenone with Weak π···π Interactions. Eur. J. Inorg. Chem..

[57] Pariyar A., Vijaykumar G., Bhunia M., Dey S.K., Singh S.K., Kurungot S., Mandal S.K. (2015). Switching Closed-Shell to Open-Shell Phenalenyl: Toward Designing Electroactive Materials. J. Am. Chem. Soc..

[58] Tan C.-M., Zhou Y.-H., Chen C.-Y., Yu J.-F., Chen K.-Q. (2016). Spin filtering and rectifying effects in the zinc methyl phenalenyl molecule between graphene nanoribbon leads. Org. Electron..

[59] Wahl I.M., Westphal E., da Costa Gouveia T.L., Sousa Santana F., Hughes D.L., Ribeiro R.R., Piccoli L.H.R., Winnischofer H. (2019). Methyl Ester Functionalized Phenalenyl Arene- and Bipyridine−Ruthenium-Based Complexes for Electroactive Langmuir−Blodgett Films. Inorg. Chem..

[60] Vijaykumar G., Bhunia M., Mandal S.K. (2019). A phenalenyl-based nickel catalyst for the hydroboration of olefins under ambient conditions. Dalton Trans..

[61] Vardhanapu P.K., Ahmed J., Jose A., Shaw B.K., Sen T.K., Mathews A.A., Mandal S.K. (2019). Phenalenyl Based Aluminum Compound for Catalytic C−H Arylation of Arene and Heteroarenes at Room Temperature. J. Org. Chem..

[62] Vijaykumar G., Pariyar A., Ahmed J., Shaw B.K., Adhikari D., Mandal S.K. (2018). Tuning the redox non-innocence of a phenalenyl ligand toward efficient nickel-assisted catalytic hydrosilylation. Chem. Sci..

[63] Chakraborty S., Ahmed J., Shaw B.K., Jose A., Mandal S.K. (2018). An Iron-Based Long-Lived Catalyst for Direct C‒H Arylation of Arenes and Heteroarenes. Chem.-Eur. J..

[64] Bellan E.V., Thevenin L., Gayet F., Fliedel C., Poli R. (2017). Catalyzed Chain Transfer in Vinyl Acetate Polymerization Mediated by 9-Oxyphenalenone Cobalt(II) Complexes. ACS Macro Lett..

[65] Bhunia M., Sahoo S.R., Shaw B.K., Vaidya S., Pariyar A., Vijaykumar G., Adhikari D., Mandal S.K. (2019). Storing redox equivalent in the phenalenyl backbone towards catalytic multi-electron reduction. Chem. Sci..

[66] Dey S.K., Mukherjee A. (2013). Zero-Order Catechol Oxidase Activity by a Mononuclear Manganese(III) Complex Showing High Turnover Comparable to Catechol Oxidase Enzyme. ChemCatChem..

[67] Das A., Ghosh T.K., Chowdhury A.D., Mobin S.M., Lahiri G.K. (2013). Electronic structure and catalytic aspects of [(trpy)(Cl)Ru(L)]_n_ incorporating potential non-innocent ligands, L^−^: 9-Oxidophenalenone and trpy: 2,2’:6’,2”-terpyridine. Polyhedron.

[68] Sen T.K., Mukherjee A., Modak A., Mandal S.K., Koley D. (2013). Substitution effect on phenalenyl backbone in the rate of organozinc catalyzed ROP of cyclic esters. Dalton Trans..

[69] Mukherjee A., Sen T.K., Ghorai P.K., Samuel P.P., Schulzke C., Mandal S.K. (2012). Phenalenyl-Based Organozinc Catalysts for Intramolecular Hydroamination Reactions: A Combined Catalytic, Kinetic, and Mechanistic Investigation of the Catalytic Cycle. Chem.-Eur. J..

[70] Ahmed J., Sreejyothi P., Vijaykumar G., Jose A., Raj M., Mandal S.K. (2017). A new face of phenalenyl-based radicals in the transition metal-free C–H arylation of heteroarenes at room temperature: Trapping the radical initiator via C–C σ-bond formation. Chem. Sci..

[71] Kim H.-S., Chung T.D., Kim H. (2001). Voltammetric determination of the p*K*a of various acids in polar aprotic solvents using 1,4-benzoquinone. J. Electroanal. Chem..

[72] Small D., Zaitsev V., Jung Y., Rosokha S.V., Head-Gordon M., Kochi J.K. (2004). Intermolecular π-to-π Bonding between Stacked Aromatic Dyads. Experimental and Theoretical Binding Energies and Near-IR Optical Transitions for Phenalenyl Radical/Radical versus Radical/Cation Dimerizations. J. Am. Chem. Soc..

[73] Caes B., Jensen D. (2008). Synthesis and Characterization of 9-Hydroxyphenalenone Using 2D NMR Techniques. J. Chem. Educ..

[74] Baker T.M., Howard K.M., Brennessel W.W., Neidig M.L. (2015). Crystal structure of a third polymorph of tris(acetylacetonato-κ^2^-*O:O’*)iron(III). Acta Cryst. Sect. E Struct. Rep. Online.

[75] Bullen G.J., Mason R., Pauling P. (1965). The Crystal and Molecular Structure of Bis(acetylacetonato)nickel(II). Inorg. Chem..

[76] Mehrotra R.C., Bohra R., Gaur D.P. (1978). Metal ß-Diketones and Allied Derivatives.

[77] Kessler V.G., Spijksma G.I., Seisenbaeva G.A., Hakansson S., Blank D.H.A., Bouvmeester H.J.M. (2006). New insight in the role of modifying ligands in the sol-gel processing of metal alkoxide precursors: A possibility to approach new classes of materials. J. Sol-Gel Sci. Technol..

[78] Montgomery H., Lingafaelter E.C. (1964). The crystal structure of diaquobisacetylacetonatonickel(II). Acta Cryst..

[79] Kudrat-E-Zahan M., Nishida Y., Sakiyama H. (2010). Identification of *cis*/*trans* isomers of bis(acetylacetonato)nickel(II) complexes in solution based on electronic spectra. Inorg. Chim. Acta.

[80] Tahir A.A., Hamid M., Zeller M., Mazhar M., Hunter A.D. (2007). Bis(µ-acetylacetonato-κ^2^-*O:O*’)bis[(acetylacetonato-κ^2^-*O:O’*)aquanickel(II)] hemihydrate. Acta Cryst. Sect. E Struct. Rep. Online.

[81] Darensbourg M., Buonomo R.M., Reibenspies J.H. (1995). Crystal structure of tetrakis[(µ^3^-methoxo-2,4-pentanedionatomethanolnickel(II)], C_28_H_56_Ni_4_O. Z. Kristallogr..

[82] El Fallah M.S., Rentschler E., Caneschi A., Gatteschi D. (1996). Synthesis, crystal structure and magnetic properties of the tetranuclear complex [Ni_4_(OCH_3_)_4_(dbm)_4_(CH_3_OH)_4_]_2_·(C_2_H_5_)_2_O. Inorg. Chim. Acta.

[83] Worthy A., Grosjean A., Pfrunder M.C., Xu Y., Yan C., Edwards G., Clegg J.K., McMurtrie J.C. (2018). Atomic resolution of structural changes in elastic crystals of copper(II) acetylacetonate. Nat. Chem..

[84] Moreno Y., Arrue R., Saavedra R., Pivan J.-Y., Pena O., Roisnell T. (2013). Magnetic and Structural Study of Unsolvated [Cu(acac)_2_], (acac = acetylacetonate). J. Chil. Chem. Soc..

[85] Ritterskamp N., Sharples K., Richards E., Folli A., Chiesa M., Platts J.A., Murphy D.M. (2017). Understanding the Coordination Modes of [Cu(acac)_2_(imidazole)_n=1,2_] Adducts by EPR, ENDOR, HYSCORE, and DFT Analysis. Inorg. Chem..

[86] Carter E., Sharples K.M., Platts J.A., Murphy D.M. (2015). Structure determination of bound nitrogen-based adducts with copper(II) acetylacetonato; an EPR, ENDOR and DFT study. Phys. Chem. Chem. Phys..

[87] Butera R.A., Waldeck D.H. (2000). An EPR Experiment for the Undergraduate Physical Chemistry Laboratory. J. Chem. Educ..

[88] Yordanov N.D., Shopov D. (1976). EPR Spectra of Mixed-Ligand Copper(II) Complexes in Solution. J. Inorg. Nucl. Chem..

[89] Nunes P., Nagy N.V., Alegria E.C.B.A., Pombeiro A.J.L., Correia I. (2014). The solvation and electrochemical behavior of copper acetylacetonate complexes in ionic liquids. J. Mol. Struct..

[90] Collison D., Powell A.K. (1990). Electron Spin Resonance Studies of “FeO_6_” Tris Chelate Complexes: Models for the Effects of Zero-Field Splitting in Distorted *S* = 5/2 Spin Systems. Inorg. Chem..

[91] Hedewy S., Hoffmann K. (1986). Electron Paramagnetic Resonance of Ferric Acetyl Acetonate. Phys. Status Solidi A.

[92] Mohammadnezhad G., Amirian A.A., Görls H., Plass W., Sandleben A., Schäfer S., Klein A. (2021). Redox Instability of Copper(II) Complexes of a Triazine-Based PNP Pincer. Eur. J. Inorg. Chem..

[93] Bubrin M., Kvapilová H., Fiedler J., Ehret F., Záliš S., Kaim W. (2018). Hybrid α-Diimine/Bis(chalcogenoether) Ligands for Copper(I) and Copper(II) Complexes. Z. Anorg. Allg. Chem..

[94] Butsch B., Klein A., Nitsche S., Stirnat K., Hawkett J.R., McInnes E.J.L., Bauer M. (2012). Generation and characterisation of the phenoxyl-radical containing Cu(II) complex [Cu(triaz)_2_]^+^ (triaz^−^ = *O*,*N* chelating triazole-phenolate). Dalton Trans..

[95] Nagao H., Komeda N., Mukaida M., Suzuki M., Tanaka K. (1996). Structural and Electrochemical Comparison of Copper(II) Complexes with Tripodal Ligands. Inorg. Chem..

[96] Pladzyk A., Ponikiewski L., Dołega A., Słowy K., Sokołowska A., Dziubinska K., Hnatejko Z. (2015). Structural Variety of Cobalt(II), Nickel(II), Zinc(II), and Cadmium(II) Complexes with 4,4’-Azopyridine:Synthesis, Structure and Luminescence Properties. Chem.-Asian J..

[97] Brahma S., Shivashankar A. (2015). Zinc acetylacetonate hydrate adducted with nitrogen donor ligands: Synthesis, spectroscopic characterization, and thermal analysis. J. Mol. Struct..

[98] Carlotto S., Floreano L., Cossaro A., Dominguez M., Rancan M., Sambia M., Casarin M. (2017). The electronic properties of three popular high spin complexes [TM(acac)_3_, TM = Cr, Mn, and Fe] revisited: An experimental and theoretical study. Phys. Chem. Chem. Phys..

[99] Schäfer S., Ott I., Gust R., Sheldrick W.S. (2007). Influence of the Polypyridyl (pp) Ligand Size on the DNA Binding Properties, Cytotoxicity and Cellular Uptake of Organoruthenium(II) Complexes of the Type [(η^6^-C_6_Me_6_)Ru(L)(pp)]^n+^ [L = Cl, n = 1; L = (NH_2_)_2_CS, n = 2]. Eur. J. Inorg. Chem..

[100] Duling D.R. (1994). Simulation of Multiple Isotropic Spin-Trap EPR Spectra (PEST Winsim). J. Magn. Reson. B.

[101] Kaim W., Fiedler J. (2009). Spectroelectrochemistry: The best of two worlds. Chem. Soc. Rev..

[102] Kaim W., Klein A. (2008). Spectroelectrochemistry.

[103] Sheldrick G.M. (2015). ShelXT—Integrated space-group and crystal-structure determination. Acta Crystallogr. Sect. A Found. Crystallogr..

[104] Sheldrick G.M. (2017). SHELXL-2017/1, Program for the Solution of Crystal Structures.

[105] Sheldrick G.M. (2015). Crystal structure refinement with SHELXL. Acta Crystallogr. Sect. C Struct. Chem..

[106] (2005). STOE X-RED. Data Reduction Program, Version 1.31/Windows.

[107] (1999). STOE X-SHAPE. Crystal Optimisation for Numerical Absorption Correction, Version 1.06/Windows.

[108] Haddon R.C., Rayford R., Hirani A.M. (1981). 2-Methyl- and 5-methyl-9-hydroxyphenalenone. J. Org. Chem..

[109] (1975). Gmelins Handbuch der Anorganischen Chemie.

