# Supplementary material for: FeIII, CuII and ZnII Complexes of the Rigid 9-Oxido-phenalenone Ligand—Spectroscopy, Electrochemistry, and Cytotoxic Properties
Source: Int J Mol Sci. 2021 Apr 12;22(8):3976. doi: 10.3390/ijms22083976 (PMC8070161; doi:10.3390/ijms22083976)
Supplement: Supplementary file 1 [file ijms-22-03976-s001.pdf]

Supplementary Material

for

Fe^{III}, Cu^{II} and Zn^{II} Complexes of the Rigid 9-Oxido-phenalenone Ligand – Spectroscopy, Electrochemistry and Cytotoxic Properties

Katharina Butsch, Alexander Haseloer, Simon Schmitz, Ingo Ott, Julia Schur, Axel Klein*

contents

Figure S1. View on the crystal structure of [Fe(opo)$_3$] along the crystallographic *a* and *b* axes.
Figure S2. π-stacking interactions in the crystal of [Fe(opo)$_3$].
Figure S3. Further π-stacking in the crystal of [Fe(opo)$_3$].
Figure S4. X-band EPR spectrum of [Cu(opo)$_2$] in DMF at 298 K with simulation.
Figure S5. X-band EPR spectrum of solid [Fe(opo)$_3$] at 298 K.
Figure S6. Cyclic voltammogramms of Hopo measured in DMF/nBu$_4$NPF$_6$ at 298 K and at 273 K.
Figure S7. UV-vis absorption spectrum of [Zn(opo)$_2$] in MeOH.
Figure S8. UV-vis absorption spectrum of [Cu(opo)$_2$] in DMF.
Figure S9. UV-vis absorption spectrum of [Fe(opo)$_3$] in DMF.
Figure S10. Absorption spectra of [Zn(opo)$_2$] recorded during reduction at −0.7 V and at –1.3 V in DMF/^{n}Bu$_4$NPF$_6$ solution.
Figure S11. Absorption spectra of [Zn(opo)$_2$] recorded during reduction at –2.0 V in DMF/^{n}Bu$_4$NPF$_6$ solution.

Table S1. Crystallographic and structure refinement data of [Fe(opo)$_3$].
Table S2. Selected metrical data of [Fe(opo)$_3$].

Crystal structure of [Ni$_4$(OCH$_3$)$_4$(acac)$_4$(CH$_3$OH)$_4$].
Figure S11. ORTEP-representations of [Ni$_4$(OCH$_3$)$_4$(acac)$_4$(CH$_3$OH)$_4$].
Table S3. Selected distances and angles of the two cluster type compounds.
Table S4. FIR vibration frequencies for the nickel cluster and [Ni(acac)$_2$].

References

Supporting Figures

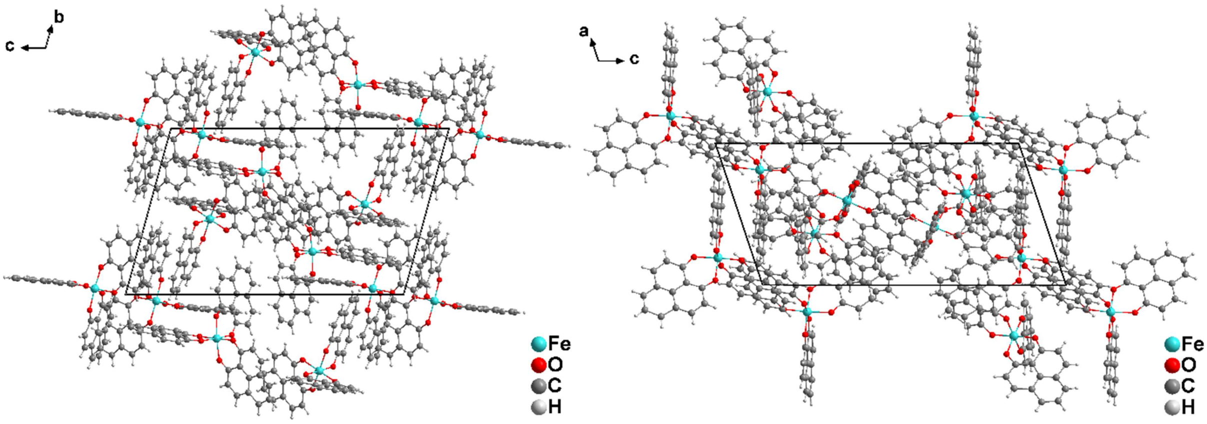

Figure S1. View on the crystal structure of [Fe(opo)3] along the crystallographic *a* (left) and *b* axes (right).

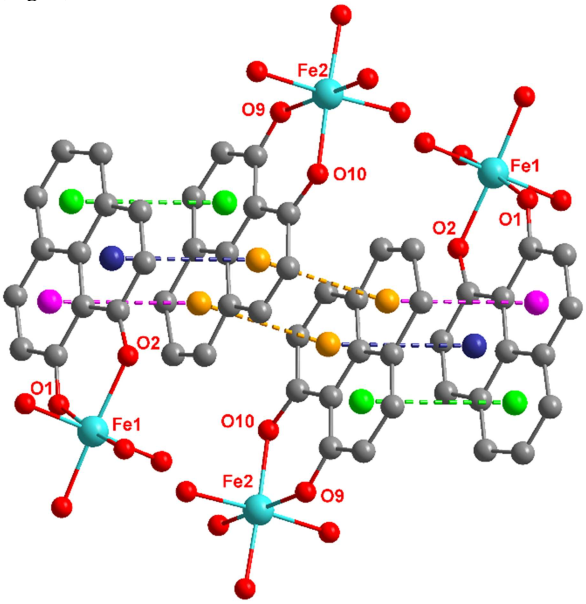

Figure S2. π-stacking interactions in the crystal of [Fe(opo)3]. Colour code: green: 3.8479(11) Å; dark blue: 3.6718(10) Å; violet: 3.7362(11) Å; yellow: 3.5366(9) Å, the coloured balls in the centre of the arene rings represent the calculated centroids.

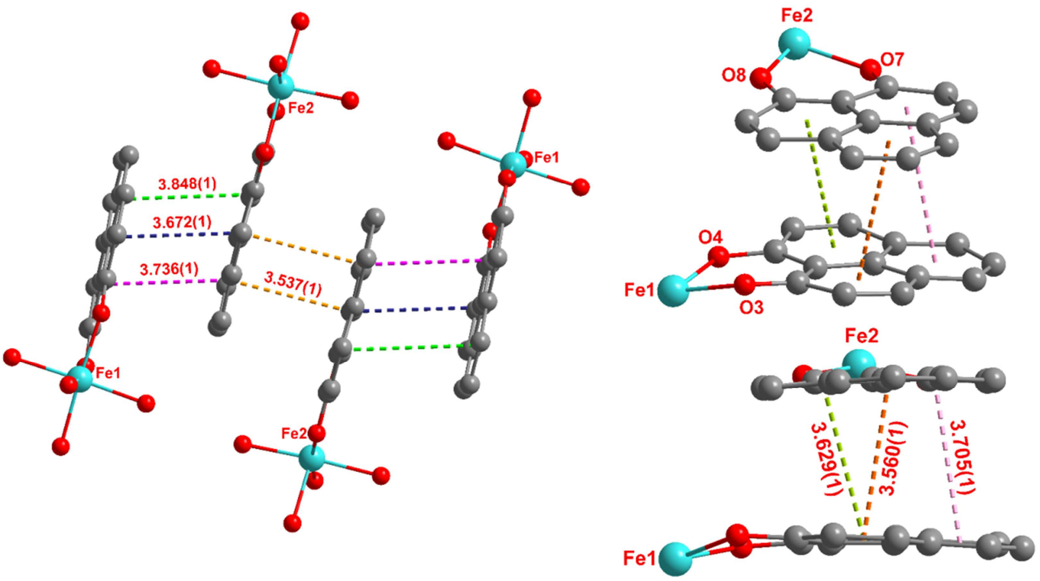

Figure S3. Further π-stacking in the crystal of [Fe(opo)3]. Colour code: green/lime: 3.6287(12) Å;

orange: 3.5597(10) Å; pink: 3.7050(9) Å.

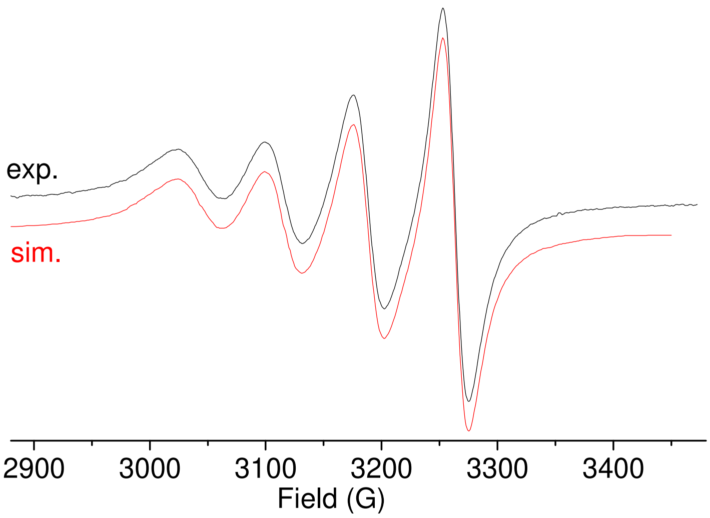

Figure S4. X-band EPR spectrum of [Cu(opo)$_2$] in DMF at 298 K. Experimental spectrum in black with simulation (in red) using: A_{Cu} = 76 G, g = 2.127. Frequency = 9.442220 GHz.

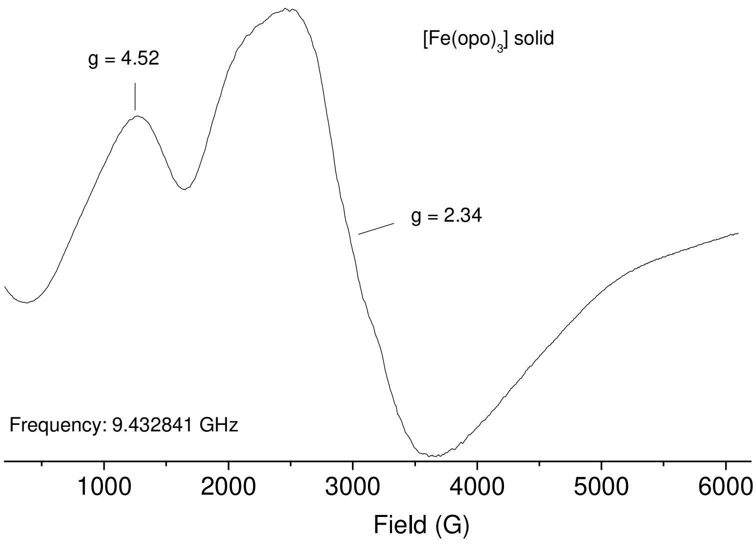

Figure S5. X-band EPR spectrum of solid [Fe(opo)$_3$] at 298 K.

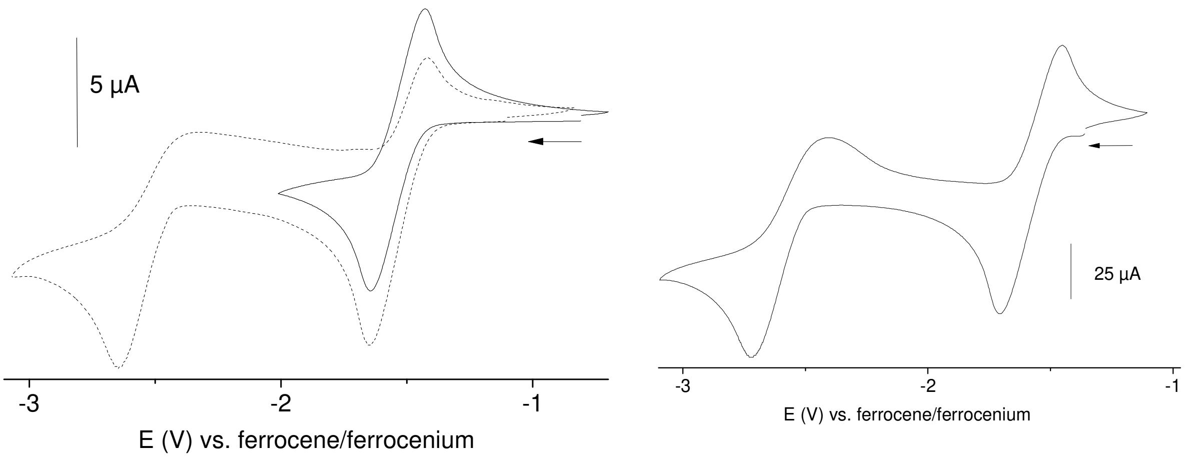

Figure S6. Cyclic voltammogramms of Hopo measured in DMF/nBu$_4$NPF$_6$ at 298 K (left) and at 273 K (right).

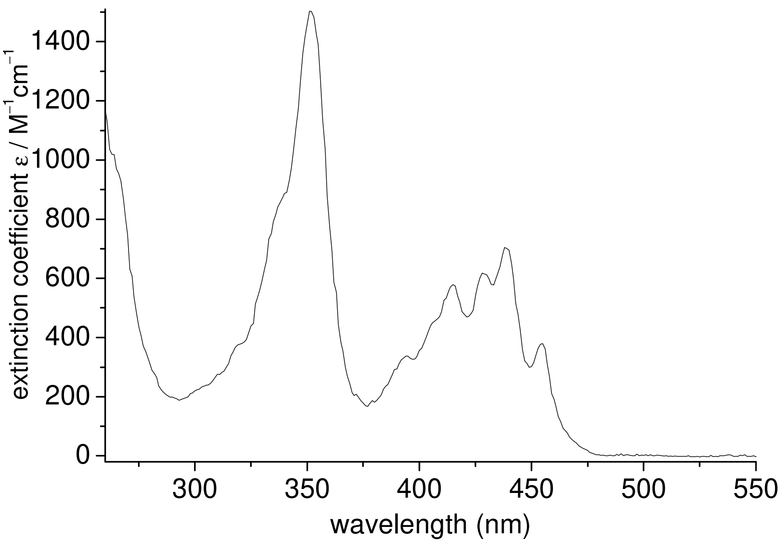

Figure S7. UV-vis absorption spectrum of [Zn(opo)$_2$] in MeOH.

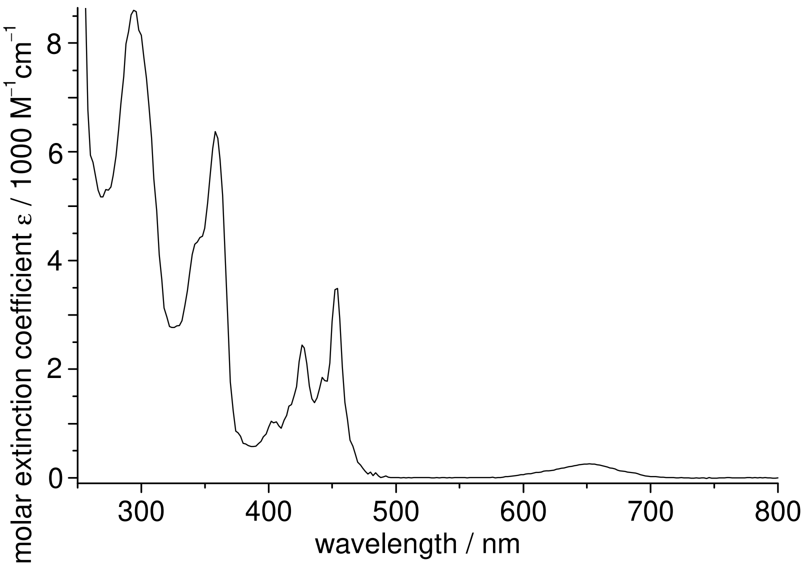

Figure S8. UV-vis absorption spectrum of [Cu(opo)$_2$] in DMF.

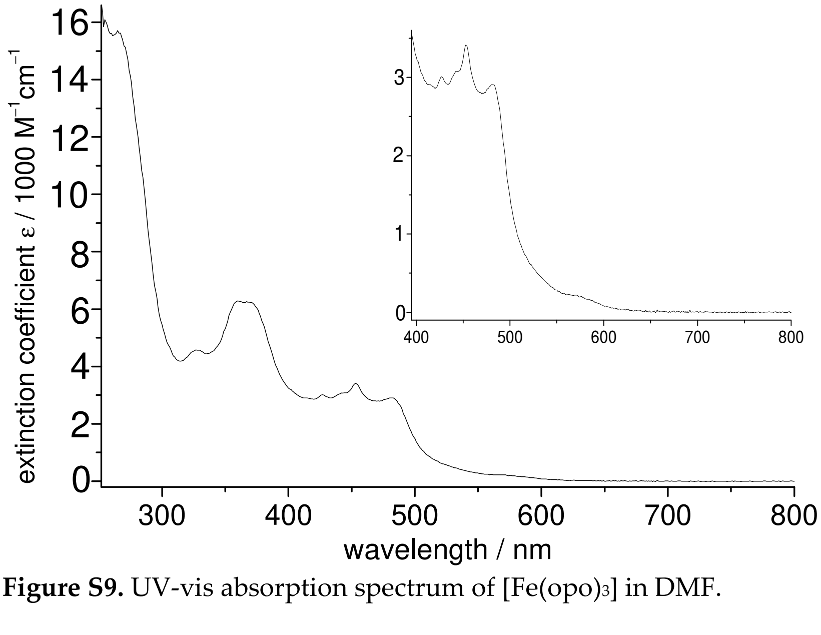

Figure S9. UV-vis absorption spectrum of [Fe(opo)$_3$] in DMF.

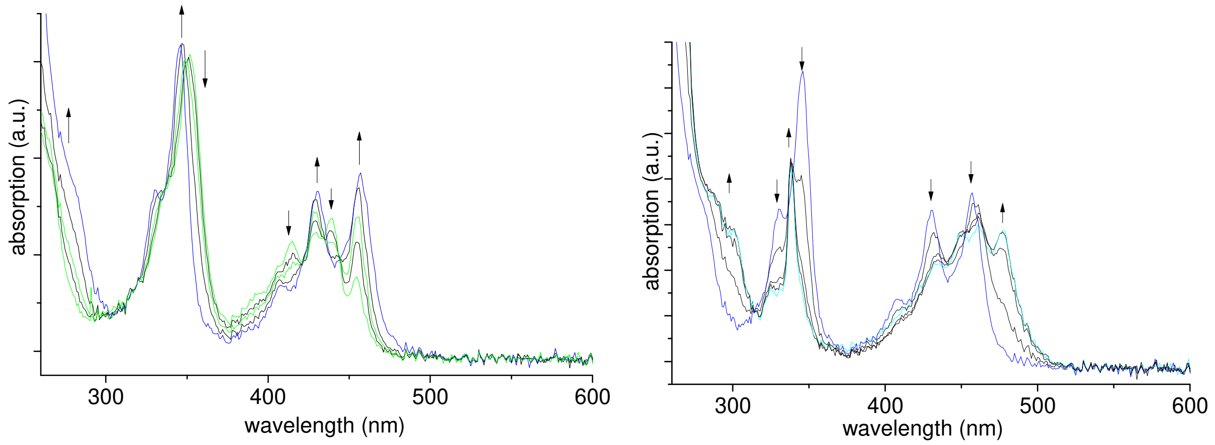

Figure S10. Absorption spectra of [Zn(opo)$_2$] recorded during reduction at −0.7 V (left) and at −1.3 V (right) in DMF/^{n}Bu$_4$NPF$_6$ solution.

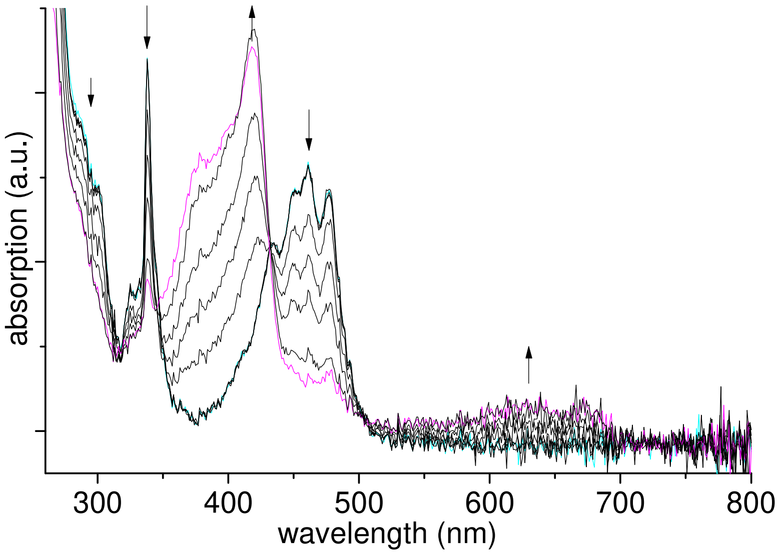

Figure S11. Absorption spectra of [Zn(opo)$_2$] recorded during reduction at –2.0 V in DMF/^{n}Bu$_4$NPF$_6$ solution.

Supporting Tables

Table S1. Crystallographic and structure refinement data of [Fe(opo)$_3$] [a]

compound	
Formula	$C_{39}H_{21}FeO_6$
Weight (g·mol^{-1})	641.41
Crystal shape	brown fraction
Crystal system	triclinic
Space group	*P*-1 (No. 2)
Temperature (K)	293(2)
Cell *a* (Å)	13.282(3)
b (Å)	16.451(3)
c (Å)	25.214(4)
..........α	98.47(2)
..........β	103.63(2)
..........γ	112.28(2)
V (Å^3) / *Z*	4779(2) / 6

ρ_{calc} (g cm^{-3})	1.337
μ (mm^{-1}) / *F(000)*	0.521 / 1974
Limiting indices	–17 < h < 17, –21< k < 21, –32 < l < 33
Refl. collected / unique	46307 / 21537
R_{int}	0.3605
Data / restraints / parameters	21537 / 0 / 1243
Goof. on F^2	0.903
Final R_1, wR_2 indices [I>2σ(I)]	R_1 = 0.0965, wR_2 = 0.2491
R_1, wR_2 (all data)	R_1 = 0.4443, wR_2 = 0.3747
Largest diff. peak and hole (e·Å^{-3})	1.62 and –0.492
CCDC	2071044

[a] Radiation wavelength λ = 0.71073 Å; Refinement method: Full-matrix least-squares on F^2.

Table S2. Selected metrical data of [Fe(opo)$_3$].

distances / Å		**molecule 1**			
Fe(1)–O(1)	1.95(1)	Fe(1)–O(6)	1.98(1)	Fe(1)–O(5)	1.99(1)
Fe(1)–O(4)	1.98(1)	Fe(1)–O(3)	1.98(1)	Fe(1)–O(2)	1.99(1)
angles / °					
O(1)–Fe(1)–O(4)	177.6(5)	O(1)–Fe(1)–O(6)	90.9(5)	O(4)–Fe(1)–O(6)	87.9(5)
O(1)–Fe(1)–O(3)	94.3(5)	O(4)–Fe(1)–O(3)	87.2(4)	O(6)–Fe(1)–O(3)	172.7(5)
O(1)–Fe(1)–O(5)	91.8(5)	O(4)–Fe(1)–O(5)	90.3(5)	O(6)–Fe(1)–O(5)	86.1(4)
O(3)–Fe(1)–O(5)	88.5(5)	O(1)–Fe(1)–O(2)	87.0(5)	O(4)–Fe(1)–O(2)	90.9(5)
O(6)–Fe(1)–O(2)	91.2(4)	O(3)–Fe(1)–O(2)	94.2(4)	O(5)–Fe(1)–O(2)	177.1(5)
C(11)–O(1)–Fe(1)	129(1)	C(1)–O(2)–Fe(1)	128(1)	C(14)–O(3)–Fe(1)	130(1)
C(24)–O(4)–Fe(1)	129(1)	C(27)–O(5)–Fe(1)	130(1)	C(37)–O(6)–Fe(1)	129(1)
distances / Å		**molecule 2**			
Fe(2)–O(12)	1.97(1)	Fe(2)–O(9)	1.99(1)	Fe(2)–O(10)	2.01(1)
Fe(2)–O(7)	1.98(1)	Fe(2)–O(11)	2.00(1)	Fe(2)–O(8)	1.99(1)
angles / °					
O(12)–Fe(2)–O(7)	95.1(5)	O(12)–Fe(2)–O(8)	94.4(5)	O(7)–Fe(2)–O(8)	86.0(5)
O(12)–Fe(2)–O(9)	172.9(5)	O(7)–Fe(2)–O(9)	91.0(5)	O(8)–Fe(2)–O(9)	89.6(5)
O(12)–Fe(2)–O(11)	85.9(5)	O(7)–Fe(2)–O(11)	90.7(5)	O(8)–Fe(2)–O(11)	176.8(5)
O(9)–Fe(2)–O(11)	90.4(5)	O(12)–Fe(2)–O(10)	87.4(5)	O(7)–Fe(2)–O(10)	176.2(6)
O(8)–Fe(2)–O(10)	90.9(5)	O(9)–Fe(2)–O(10)	86.7(5)	O(11)–Fe(2)–O(10)	92.3(5)
distances / Å		**molecule 3**			
Fe(3)–O(18)	1.94(1)	Fe(3)–O(15)	1.96(1)	Fe(3)–O(14)	1.97(1)
Fe(3)–O(13)	2.00(1)	Fe(3)–O(17)	1.98(1)	Fe(3)–O(16)	2.01(1)
angles / °					
O(18)–Fe(3)–O(15)	175.7(5)	O(18)–Fe(3)–O(14)	93.5(5)	O(15)–Fe(3)–O(14)	88.5(5)
O(18)–Fe(3)–O(17)	87.2(5)	O(15)–Fe(3)–O(17)	88.9(5)	O(14)–Fe(3)–O(17)	90.3(5)
O(18)–Fe(3)–O(13)	93.4(5)	O(15)–Fe(3)–O(13)	90.5(5)	O(14)–Fe(3)–O(13)	86.3(5)
O(17)–Fe(3)–O(13)	176.6(5)	O(18)–Fe(3)–O(16)	90.9(5)	O(15)–Fe(3)–O(16)	87.4(5)
O(14)–Fe(3)–O(16)	174.7(5)	O(17)–Fe(3)–O(16)	92.9(5)	O(13)–Fe(3)–O(16)	90.5(5)

Crystal structure of [$Ni_4(OCH_3)_4(acac)_4(CH_3OH)_4$].

Polygonal green crystals of [$Ni_4(OCH_3)_4(acac)_4(CH_3OH)_4$] were obtained from the reaction solution of [$Ni(acac)_2$] and Hopo. The structure was solved and refined in the monoclinic space group *C*2/c. The same structure was already reported by Reibenspeis *et al.*[1] The molecular structure of the cubane like cluster is shown in Figure S11.

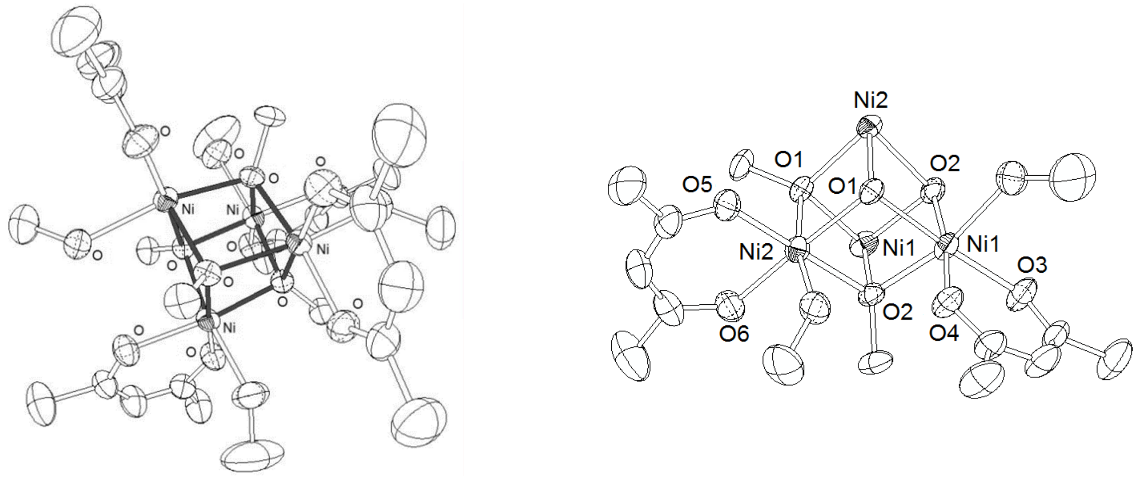

Figure S11. ORTEP-representations (50% probability level) of the Ni–O hetero-cubane cluster [$Ni_4(OCH_3)_4(acac)_4(CH_3OH)_4$]. Left: view on the complete cluster molecule, right: hetero-cubane with numbering; H atoms were omitted for clarity.

[$Ni_4(OCH_3)_4(acac)_4(CH_3OH)_4$] is a derivative of [$Ni_4(OCH_3)_4(dbm)_4(CH_3OH)_4]_2·Et_2O$ (dbm = dibenzoylmethane) published by Gatteschi *et al.* (Table S3).[2]

Table S3. Selected distances and angles of the two cluster type compounds

	[$Ni_4(OCH_3)_4$ $(acac)_4$ $(CH_3OH)_4$]	[$Ni_4(OCH_3)_4$ $(dbm)_4$ $(CH_3OH)_4]_2·Et_2O$		[$Ni_4(OCH_3)_4$ $(acac)_4$ $(CH_3OH)_4$]	[$Ni_4(OCH_3)_4$ $(dbm)_4$ $(CH_3OH)_4]_2·Et_2O$
Ni–Ni-1	3.0645(8)	3.048(3)	Ni–$O_{methoxide}$-2	2.0620(9)	2.072(9)
Ni–Ni-3	3.1146(8)	3.111(3)	Ni–$O_{chelate}$-1	2.0047(9)	1.975(9)
Ni–$O_{methanol}$-1	2.1137(6)	2.127(9)	Ni–$O_{chelate}$-2	2.0265(7)	2.033(9)
Ni–$O_{methanol}$-2	2.1452(8)	2.16(1)	Ni–O–Ni-1	80.77(1)	95.8(4)
Ni–$O_{methoxide}$-1	2.0404(6)	2.02(1)	Ni–O–Ni-2	98.99(2)	100.3(4)

In contrast to [$Ni_4(OCH_3)_4(dbm)_4(CH_3OH)_4]_2·Et_2O$, [$Ni_4(OCH_3)_4(acac)_4(CH_3OH)_4$] is highly air stable. Freshly prepared crystals of the latter compound were analysed by far infrared spectroscopy as well as crystals stored six months exposed to air (crystals became amorphous powders after three to four months) yielding no differences of IR frequencies (Table S4)[3].

Table S4. FIR vibration frequencies for the nickel cluster and [$Ni(acac)_2$] [a]

[$Ni_4(OCH_3)_4(acac)_4(CH_3OH)_4$]	[$Ni(acac)_2$]	assignment [3]
677	666	$\delta_{ring\ deformation}$ + ν(M–O)
657	-	-
573	579	ν(M–O)
564	563	ν(M–O)
449	452	δ (C–CHO) + ν(M–O)
420	427	δ (O–M–O)
337	-	-
290	-	-
250	-	-

[a] Resonances in cm^{-1}.

References:

(1) M. Darensbourg, R. M. Buonomo, J. H. Reibenspies, Crystal structure of tetrakis[(μ^3-methoxo-2,4-pentanedionatomethanolnickel(II)], $C_{28}H_{56}Ni_4O$. *Z. Kristallogr.* **1995**, *210*, 469−451.

(2) M. S. El Fallah, E. Rentschler, A. Caneschi, D. Gatteschi, Synthesis, crystal structure and magnetic properties of the tetranuclear complex $[Ni_4(OCH_3)_4(dbm)_4(CH_3OH)_4]_2 \cdot (C_2H_5)_2O$. *Inorg. Chim. Acta* **1996**, *247*, 231−235.

(3) A. M. A. Bennett, G. A. Foulds, D. A. Thornton, The IR and ^{1}H, ^{13}C NMR Spectra of the Nickel(II), Copper(II) and Zinc(II) Complexes of 2,4-Pentanedione, 4-Imino-2-pentanone, 4-Thioxo-2-pentanone and 2,4-Pentaedithione: A Comparative Study. *Polyhedron* **1989**, *8*, 2305–2311.
